# Nonfucosylation of an anti-TIGIT antibody enhances FcγR engagement, driving innate immune activation and antitumor activity

**DOI:** 10.3389/fimmu.2023.1280986

**Published:** 2023-11-01

**Authors:** Alyson J. Smith, Robert E. Thurman, Weiping Zeng, Bryan Grogan, Sasha Lucas, Guadalupe Gutierrez, Ryan A. Heiser, Serena W. Wo, Amber Blackmarr, Scott Peterson, Shyra J. Gardai

**Affiliations:** Research Department, Seagen, Bothell, WA, United States

**Keywords:** TIGIT, anti-TIGIT antibody, nonfucosylation, antitumor, FcγR-enhanced antibody

## Abstract

TIGIT is an immune checkpoint receptor expressed on activated and memory T cells, immunosuppressive T regulatory cells, and natural killer (NK) cells. TIGIT has emerged as an attractive target for antitumor therapies, due to its proposed immunosuppressive effects on lymphocyte function and T cell activation. We generated an anti-TIGIT monoclonal antibody (mAb) that binds with high affinity to human, non-human primate, and murine TIGIT and through multiple experimental methodologies demonstrated that checkpoint blockade alone is insufficient for antitumor activity. Generating anti-TIGIT mAbs with various Fc backbones we show that muting the Fc-Fcγ receptor (FcγR) interaction failed to drive antitumor activity, while mAbs with Fc functional backbones demonstrate substantial antitumor activity, mediated through activation of antigen-presenting cells (APCs), T cell priming, and NK-mediated depletion of suppressive Tregs and exhausted T cells. Further, nonfucosylation of the Fc backbone resulted in enhanced immune responses and antitumor activity relative to the intact IgG1 backbone. The improved activity correlated with the biased FcγR interaction profile of the nonfucosylated anti-TIGIT mAb, which supports that FcγRIIIa binding with decreased FcγRIIb binding favorably activates APCs and enhances tumor-specific CD8^+^ T cell responses. The anti-TIGIT mAbs with intact FcγR interacting backbones also demonstrated synergistic enhancement of other standard antitumor treatments, including anti-PD-1 treatment and a model monomethyl auristatin E antibody–drug conjugate. These findings highlight the importance of the anti-TIGIT mAb’s Fc backbone to its antitumor activity and the extent to which this activity can be enhanced through nonfucosylation of the backbone.

## Introduction

Adaptive immune checkpoint blockade to release inhibited immune cells and drive antitumor activity has become a mainstay of clinical cancer therapy. The most clinically successful class of checkpoint inhibitor therapies are antibodies targeting the programmed cell death protein 1/programmed-death ligand 1 (PD-1/PD-L1) axis, which have shown benefits to overall survival in multiple tumor types. While many patients treated with anti-PD-1/PD-L1 targeted drugs can achieve clinically meaningful responses, the majority do not. This raises the possibility that other immune modalities, including other nonredundant checkpoints, may also restrict tumor immunity and therefore are potential targets for anticancer therapies. Other inhibitory immune receptors of recent interest include T cell immunoglobulin mucin domain 3 (TIM-3), lymphocyte-activation gene 3 (LAG3), cytotoxic T lymphocyte-associated protein 4 (CTLA-4) and T cell immunoreceptor with immunoglobulin (Ig) and immunoreceptor tyrosine-based inhibitory motif (ITIM) domains (TIGIT). Therapeutic agents that target inhibitory immune receptors to promote existing T cell responses and the generation of new CD8 T cell responses may have the potential to improve clinical outcomes in cancer. For example, further activity might be derived by targeting agents that not only release these parallel immune checkpoints, but that also stimulate the generation of new, antigen-specific CD8^+^ T cell responses.

TIGIT is a member of the polio virus/nectin receptor family that was discovered in 2009 (1). It has been reported to be expressed on activated and memory T cells, immunosuppressive T regulatory cells (Tregs), and natural killer (NK) cells. TIGIT has been described as a checkpoint receptor since engagement of TIGIT with ligands CD155 and CD112 (higher and lower affinity, respectively) inhibits lymphocyte function *in vitro* (1). This engagement drives an inhibitory signal and also supplants ligand binding from the co-stimulatory receptor CD226 because the TIGIT-CD155 interaction is of higher affinity (2). CD155 is normally expressed on several types of antigen-presenting cells (APCs), but is also overexpressed in several types of cancer (3–5), which might facilitate tumor growth and immune evasion (6). TIGIT-ligand engagement can further limit T cell and innate immune cell responses through downregulation of the T cell receptor (TCR) α chain and the TCR complex (7), reduction of p-extracellular signal-related kinase signaling in T cells (8), and suppression of NK-mediated cytotoxicity (9). The ability of TIGIT to shut down lymphocyte function concomitant with sequestering ligands important for T cell activation has highlighted the therapeutic potential of targeting this receptor for T cell reactivation.

Due to the proposed immunosuppressive activity of TIGIT, there is great interest in evaluating anti-TIGIT therapeutic antibodies in clinical trials (10). Antibodies that target immune checkpoints were originally thought to function primarily by blocking the inhibitory signal on T cells and “reinvigorating” their activity. This appears to be the case for several checkpoints, such as PD-1, PD-L1, and the recently approved LAG3 therapy (11). While these approved products primarily rely on receptor blockade and contain inert crystallizable fragment (Fc) backbones, there is growing evidence that monoclonal antibodies (mAbs) targeting checkpoint receptors such as CTLA-4 and TIGIT can function through more than just simply pathway blockade. The backbones of these antibodies play a valuable role as they can elicit a range of functions that contribute to the antibody’s antitumor activity, including removal of antigen-positive cells (12–15). Specifically, these mAbs can mediate additional activities through their Fc via interactions with Fcγ receptors (FcγR) expressed on immune cells (16). These activities include antibody-dependent cellular cytotoxicity (ADCC) and/or antibody-dependent cellular phagocytosis (ADCP), initiated upon activation of these cells through the immunoreceptor tyrosine-based activation motif (ITAM) on the FcγRs (17). Importantly, though, APCs can co-express both ITAM containing activating FcγRs (I, IIa, and III) and ITIM containing inhibitory receptor (IIb), and the appropriate balance between these two divergent receptors dictates the ultimate activation state and function of the engaged APC. Exactly how these signal modulation mechanisms may contribute to the antitumor activity of anti-TIGIT mAbs remains poorly understood.

Here we report the discovery and preclinical evaluation of an anti-TIGIT mAb that binds with high affinity to human, non-human primate, and murine TIGIT. By transplanting the variable domains of this anti-TIGIT mAb onto various antibody backbones, we show that a functionally intact Fc backbone is required for the antitumor activity of the anti-TIGIT mAb across several syngeneic murine tumor models. We also demonstrate that the contribution of the intact Fc backbone to antitumor activity may occur through Fc-mediated activation of APCs. This optimal APC activation is uniquely observed when the FcγRs are triggered using a nonfucosylated Fc backbone. The superior immune activation achieved with the nonfucosylated anti-TIGIT mAb translated into enhanced antitumor T cell responses, which may support distinctive partnerships with other therapeutic modalities, including immunotherapies that can block exhaustion of the new T cells, as well as chemotherapies, such as vedotin antibody–drug conjugate (ADC) therapies, which have been established to drive immunogenic cell death and provide new tumor-specific antigens for generation of new CD8 T cells.

## Results

### TIGIT expression on terminally exhausted T cells and Tregs

**Figure 1:**
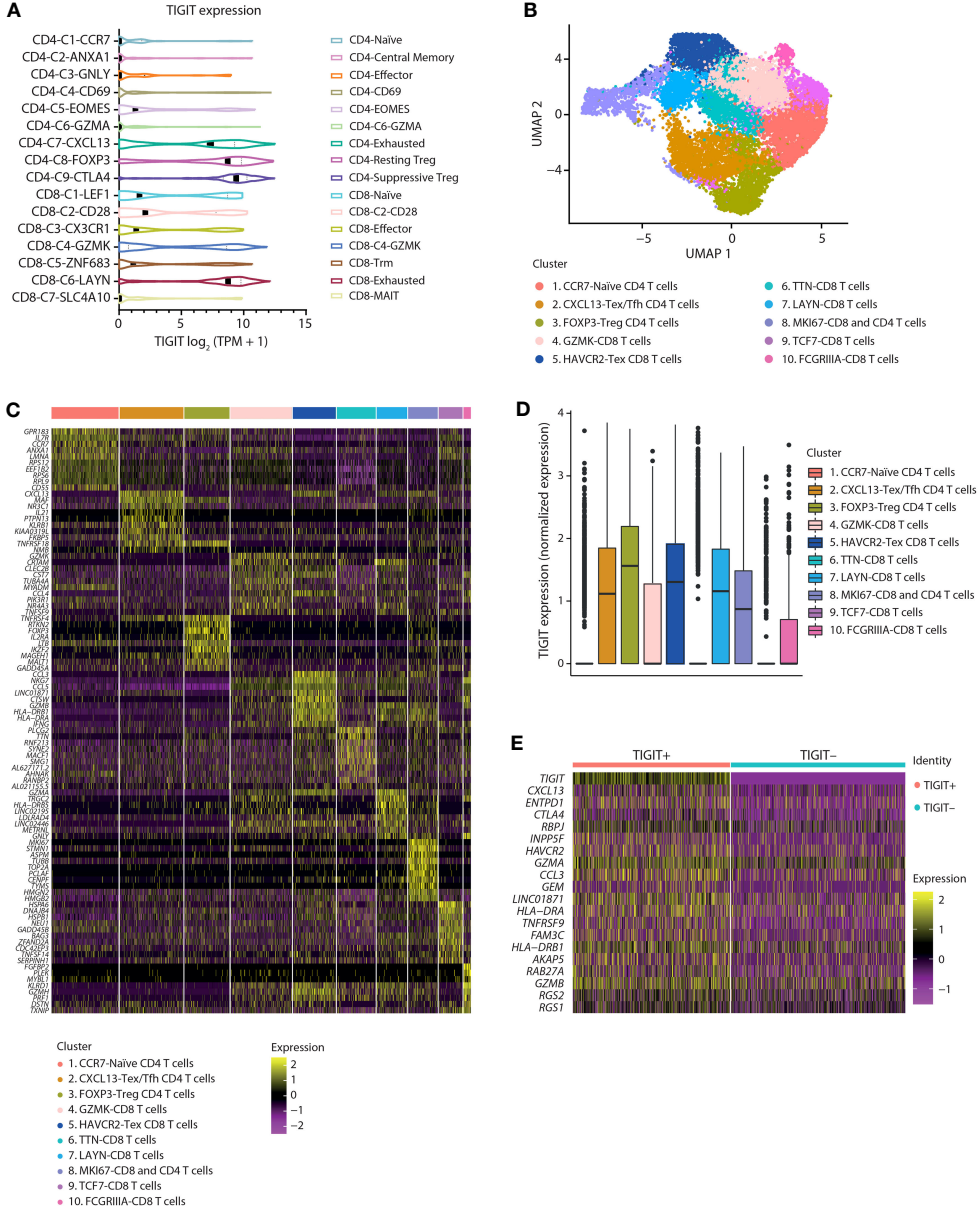
In NSCLC tumors, TIGIT expression is enriched on Tregs and dysfunctional T cells. **(A)** Violin plots showing the log_2_ (TPM + 1) expression of TIGIT transcripts across 16 T cell clusters as identified by scRNA sequencing. Data were reanalyzed from a primary data set provided by Guo et al., 2018 (18). **(B)** Identification of T cell subpopulations from separate internal scRNA sequencing of three NSCLC tumor-infiltrating lymphocytes. UMAP embeddings colored by cluster. **(C)** Heatmaps showing z-scored average expression of T cell subset marker genes ordered by significance within each cluster. **(D)** Box plot of TIGIT expression across clusters. **(E)** Heatmap of the top 20 differentially expressed genes between CD8^+^ T cells with or without TIGIT expression.

To elucidate the cellular mechanisms responsible for immune activation by anti-TIGIT antibodies, TIGIT expression on intratumoral T cells from human lung cancer tumors was assessed. Reanalysis of published single-cell RNA sequencing (scRNA-seq) data (18) of tumor-infiltrating T cells from patients with non-small cell lung cancer (NSCLC) showed TIGIT prominently expressed by resting and suppressive Tregs (CD4 subsets 8-FOXP3 and 9-CTLA-4, respectively), exhausted CD4^+^ T cells (CD4 subset C7-CXCL13), and exhausted CD8^+^ T cells (CD8 subset C6-LAYN) (**Figure 1A**). To gain further insight into the correlation between TIGIT expression with T cell phenotype in tumors, we performed independent scRNA-seq on intratumoral NSCLC T cells (**Figure 1B**). Unbiased clustering of sorted CD45^+^CD3^+^ T cells from three pretreatment NSCLC donors yielded T cell clusters characterized by marker genes generally overlapping those described in a previous single-cell characterization of T cells in NSCLC by Guo et al. (18). We identified three CD4^+^ T cell clusters consisting of naïve (Cluster 1-CCR7), Treg (Cluster 3-FOXP3), and exhausted T cell populations (Cluster 2-CXCL13). CD4^+^ exhausted T cells also displayed high expression of markers associated with T follicular helper cells (ICOS, interleukin [IL]-21, BCL6). CD8^+^ T cell clusters were broader, with a population of terminally exhausted cells enriched for CCL3, HAVCR2, LAG3, and GZMA (Cluster 5-HAVCR2). Another CD8^+^ T cell group (Cluster 6-TTN) was enriched for PLCG2, TTN, and MACF1, possibly reflecting memory T cells. Interestingly, TRGC2, a gene encoding the constant domain for the TCRγ chain, was found to be expressed by three clusters (Cluster 4-GZMK, Cluster 7-LAYN, Cluster 10-FcγRIIIa) also expressing CD8a. As the T cell co-receptors CD4 and CD8 are not expressed by bona fide γδ T cells, we consider the GZMK, LAYN, and FcγRIIIa expressing CD8^+^ T cell groups to be primarily CD8^+^ T cells with some γδ T cells included in the clusters, owing to shared cytotoxic similarities. GZMK and LAYN CD8^+^ T cell clusters shared many transcripts with the HAVCR2 CD8^+^ T cell cluster and likely reflect a continuum of activated and dysfunctional states. **Figure 1C** shows a heatmap of the most significant genes expressed in each cluster as determined by using the FindMarkers function in Seurat, which utilizes the default Wilcoxon Rank Sum test. As with Guo et al. (18), TIGIT expression was highly enriched within FOXP3 Treg, CXCL13 CD4, and HAVCR2+ exhausted CD8^+^ T cell clusters, with TIGIT-expressing cells also found in related GZMK and LAYN clusters. TIGIT expression was lowest in naïve, memory, and progenitor exhausted clusters (Clusters 1-CCR7, 6-TTN, and 9-TCF7) (**Figure 1D**). Comparing differentially expressed genes between TIGIT-expressing CD8^+^ T cells and other CD8^+^ T cells (TIGIT >0 vs TIGIT=0) showed TIGIT expression was associated with core transcripts of T cell dysfunction in tumors, including ENTPD1 (CD39), CTLA-4, RBPJ, HAVCR2 (TIM-3), GZMA, and TNFRSF9 (4-1BB) (**Figure 1E**) (19). Taken together, scRNA-seq analysis of intratumoral T cells in NSCLC shows that TIGIT expression is enriched on Tregs and dysfunctional CD4^+^ and CD8^+^ T cells in tumors.

### Generation and characterization of anti-TIGIT mAbs

**Figure 2:**
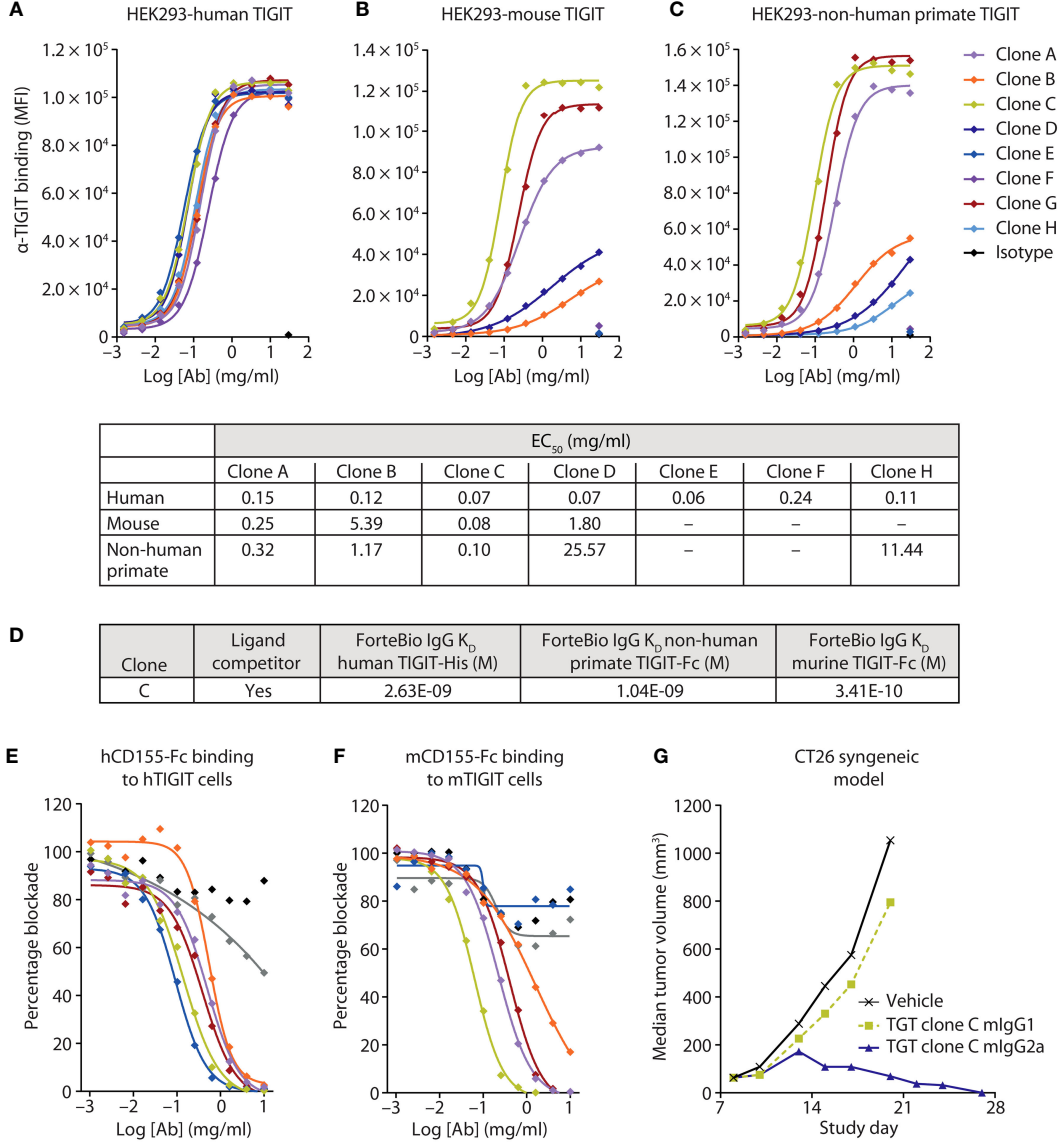
A highly potent and active human, murine, and non-human primate cross-reactive anti-TIGIT antibody was generated. **(A-C)** Several anti-TIGIT mAbs were added in increasing concentrations to human **(A)**, murine **(B)**, or non-human primate **(C)** TIGIT-expressing HEK cells and assessed for binding by flow cytometry. The strength of the interactions is denoted in the table as the half-maximal effective binding concentration (EC_50_) of each clone for each cell line. **(D)** Binding kinetics of the lead clone were assessed via Octet binding to the indicated proteins. **(E, F)** Several anti-TIGIT mAbs were added in increasing concentrations to human **(E)** or murine **(F)** expressing TIGIT cells and labeled CD155 protein. Inhibition of the CD155:TIGIT binding by these mAbs was determined by a decrease in MFI when analyzed via flow cytometry. **(G)** The lead anti-TIGIT clone was cloned onto either a murine IgG1 or murine IgG2a backbone and assessed for antitumor activity on established CT26 tumors after six doses of 5 mg/kg given every 3 days.

To accurately assess the therapeutic potential of an anti-TIGIT mAb preclinically, we generated a human, non-human primate, murine cross-reactive anti-TIGIT mAb using a yeast-based antibody presentation system (20). Characterization of the clones was performed using cell binding to human embryonic kidney (HEK) cells transduced to stably express human, murine, or non-human primate TIGIT (**Figures 2A–C**), and ForteBio binding to purified protein from the three species (**Figure 2D**). Of the 65 IgG1 clones tested, all antibodies showed specific binding to the HEK 293-hTIGIT, and 43 had an affinity <100 nM for the TIGIT monomer. Fifty-three clones specifically bound the HEK 293-non-human primate TIGIT line (**Figure 2C**, subset of clones shown), while less than half (31 antibodies) bound the HEK 293-murine TIGIT line (**Figure 2B**). Clones were also screened for the ability to block human and murine TIGIT/CD155 (**Figures 2E, F**) and hCD112 (not shown) interactions, with most clones found to be functional and similar in their ability to relieve this checkpoint. From the binding and blockade experiments, a lead anti-TIGIT mAb was selected that had roughly equivalent binding affinity for human, non-human primate, and murine TIGIT, and which was potent at relieving checkpoint blockade.

To determine the contribution of blocking activity to antitumor responses, the variable domains of the lead anti-TIGIT mAb were cloned onto a murine IgG1 (mIgG1) or murine IgG2a (mIgG2a) backbone. The mIgG1 backbone is well established as having decreased FcγR binding in the mouse and is akin to a human IgG4 backbone in terms of FcγR engagement (21), which is often used in immune checkpoint blocking antibodies to diminish the depletion of reinvigorated antigen-positive T-cells. The mIgG1 backbone primarily examines the contribution of TIGIT blockade to antitumor activity. The mIgG2a backbone engages multiple FcγRs and is used to approximate Fc interactions of a human IgG1 antibody (22). Treatment with the mIgG1 backbone anti-TIGIT mAb did not drive antitumor activity in the CT26 syngeneic murine tumor model (**Figure 2G**), despite showing robust ability to block the inhibitory TIGIT signaling axis (**Figure 2F**). However, use of the mIgG2a backbone anti-TIGIT mAb drove substantial antitumor efficacy (**Figure 2G**), suggesting an important role for FcγR engagement in anti-TIGIT activity.

### Effects of anti-TIGIT antibody Fc backbone composition on TIGIT signaling blockade and FcγR engagement

**Figure 3:**
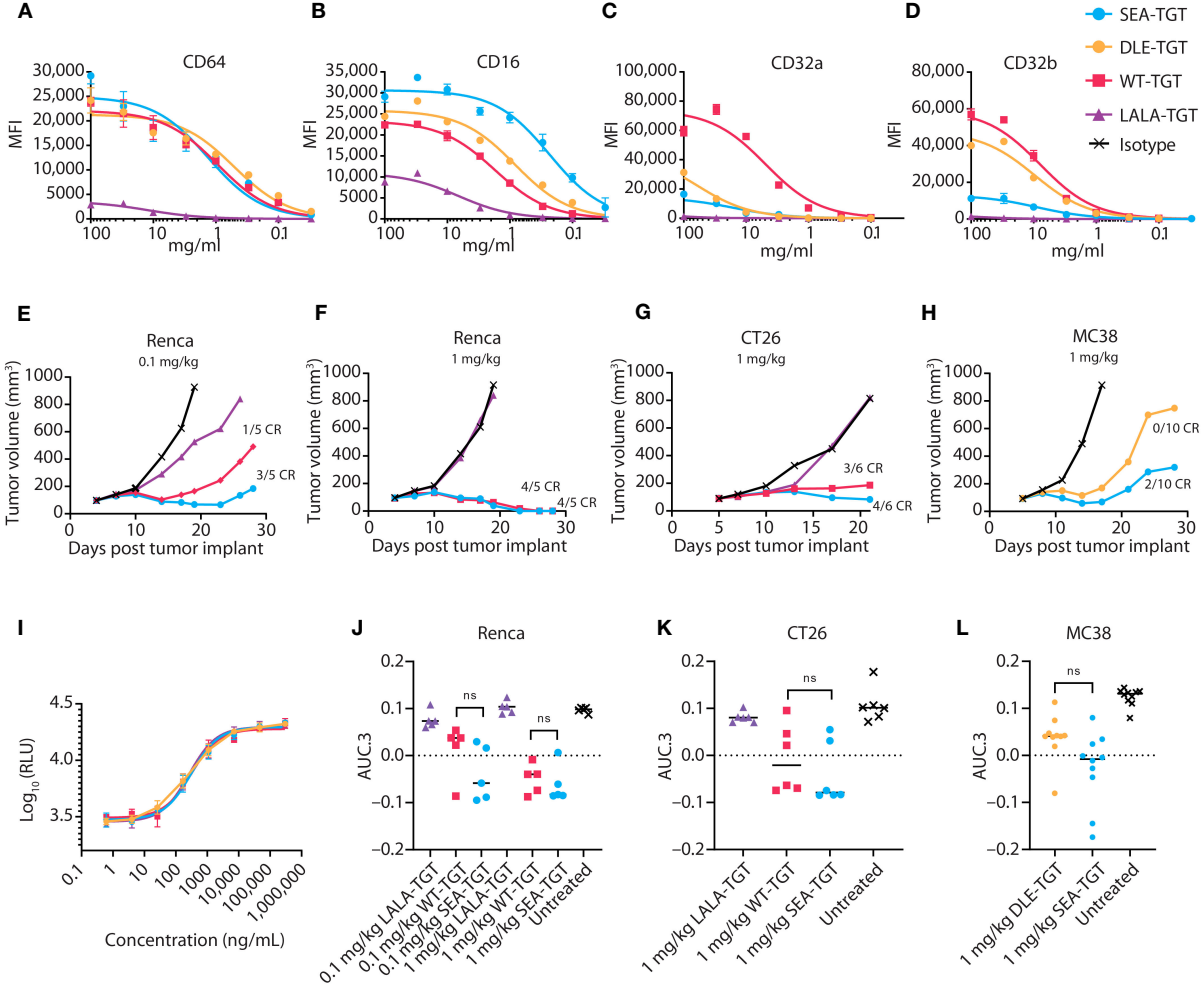
Anti-TIGIT antibodies with varying backbones equally relieve TIGIT signaling blockade, but differentially engage FcγRs, resulting in differential antitumor activity. **(A-D)** CHO cells expressing the indicated FcγR were incubated with increasing concentrations of the indicated anti-TIGIT antibody, then assessed via FACS for on-cell binding. **(E-H)** Tumor growth delay in Renca renal carcinoma **(E, F)**, CT26 colon carcinoma **(G)**, and MC38 colon carcinoma **(H)** was assessed in mice. Animals were dosed with 1 or 0.1 mg/kg of the indicated antibody as noted when tumors reached 100 mm^3^ and were followed for tumor growth over time. Median tumor volume over time is plotted. Complete regressions (CR), as denoted when mice reached 0 mm^3^ tumor volume, are noted on the graphs for each group. **(I)** Ability of the indicated anti-TIGIT mAb to block the TIGIT: CD155 axis and restore CD226 signaling in a Jurkat reporter cell line was measured via increases in luciferase production. **(J-L)** Overall antitumor activity of various TGT molecules across different syngeneic models. Antitumor activity is represented as normalized area under the tumor-response curve (AUC.3) per Guo et al. (BMC Cancer 19, 718 (2019). Tukey’s Honestly Significant Difference adjusted p-values for differences in AUC.3 for each treatment arm, represented as individual animal responses, were run and are denoted. ns, not significant.

Given the difference in antitumor activity between the mIgG1 and mIgG2a backbones, we sought to further define the role of the Fc interactions in driving antitumor activity. In addition to the wild-type, human IgG1 anti-TIGIT mAb (WT-TGT), we generated three different variants to explore the contribution of the Fc backbone to anti-TIGIT functionality. To mitigate binding to all FcγRs, an Fc-muted form of the anti-TIGIT mAb, akin to the mIgG1 antibody (**Figure 2G**) was generated through mutation of L234A/L235A/P329G residues in the backbone (referred to for simplicity as “LALA-TGT”) (23, 24). We also sought to maximize Fc-driven activity of the anti-TIGIT mAb, by generating variants that drive a range of FcγR engagement. To maximize interaction between the anti-TIGIT mAb and FcγRIIIa expressed on APCs, we utilized our proprietary sugar engineering technology to create a nonfucosylated version of the antibody, SEA-TGT. Nonfucosylated antibodies enhance FcγRIIIa interaction (25) while also limiting interactions with the inhibitory FcγRIIb receptor. To explore other reported methods of modifying FcγR interactions, the S239D/A330L/I332E (DLE) mutations were made in the backbone to generate DLE-TGT. Unlike the nonfucosylated modification, these mutations increase binding to all FcγRs, both activating and inhibitory determined both through ex vivo, cell free binding via BLI as well as on cell binding (**Table 1** and **Figures 3A–D**) (26).

**Table 1:** Affinity of various anti-TIGIT antibodies for Fcg(gamma)Rs measured via BLI.

KD(µM)	CD64	CD32a (H131)	CD32A (R131R)	CD32b	CD16 (F158)	CD16 (V158)
**WT-TGT**	0.0021	2.731	4.85	32.41	5.78	1.19
**LALA-TGT**	25.5000	No binding	No binding	No binding	No binding	No binding
**DLE-TGT**	0.0002	1.622	1.56	3.13	0.08	0.06
**SEA-TGT**	0.0019	3.216	3.64	19.08	0.49	0.09

Dissociation constants (KD) were measured via Octet binding and are denoted for each mAb for each human Fcg(gamma)R protein, with allelic variants denoted.

WT-TGT bound to all FcγRs while the Fc-muted LALA-TGT showed greatly diminished binding to all FcγRs (**Table 1** and **Figures 3A-D**) and abolished complement-dependent activity [not shown (23)]. The SEA-TGT nonfucosylation resulted in a unique FcγR interaction profile, with higher FcγRIII/CD16 binding compared with the standard IgG1 backbone antibody (WT-TGT) and concomitant diminished/low binding to the inhibitory FcγRIIb–CD32b receptor (**Table 1** and **Figures 3A–D**). SEA-TGT also showed reduced binding to FcγRIIa/CD32a (**Figure 3C**). Cell binding analyses confirmed that the DLE modification enhanced FcγRIIIa binding over the IgG1 backbone (WT-TGT) but, unlike the SEA modification, also maintained/increased FcγRIIb binding (**Table 1** and **Figures 3A–D**). As expected, changing the antibody backbone did not affect the ability of the antibodies to bind to human TIGIT or to relieve the TIGIT/CD155/CD226 signaling blockade (**Figure 3I**).

To assess how these changes in Fc backbone affect *in vivo* antitumor activity, we utilized several syngeneic models. Importantly, the trends for Fc engagement of the various mIgG2a backbones of the anti-TIGIT antibodies, i.e., higher FcγRIV/hCD16 binding for SEA and decreased FcγRIV/hCD16 binding for LALA, were similar to those observed in humans (not shown). The Fc-muted LALA-TGT, which retained complete relief of the TIGIT signaling checkpoint but was unable to engage FcγRs, did not elicit appreciable antitumor activity or curative responses in any of the syngeneic models tested, even at the highest dose of 5 mg/kg (**Figures 3E–G, J, K**; 5 mg/kg dose not shown). In contrast, all mIgG2a anti-TIGIT mAbs with intact FcγR binding (WT-TGT, SEA-TGT, and DLE-TGT) showed significant tumor growth delay in many of the disparate syngeneic models assessed. These data reaffirm that checkpoint blockade alone is not sufficient to drive preclinical antitumor activity. Demonstrating the importance of intact Fc-interactions for anticancer activity, SEA-TGT was able to control tumor growth to a similar degree as the WT-TGT (**Figure 3F**) with increased curative responses (**Figure 3G**) and trends for increased overall activity, though does not reach statistical significance, (**Figures 3J–L**) seen in multiple models. To further evaluate differences in antitumor responses between WT-TGT and SEA-TGT, antibodies were administered at lower doses in multiple syngeneic models and at these lower doses, SEA-TGT again drove a more prominent trend for enhanced antitumor activity; although tumor growth delay was not significantly different, SEA-TGT did drive three times more complete responses (**Figures 3E, J**). To investigate whether the antitumor advantage afforded by the TIGIT mAb backbone was through the preferential binding to activating FcγRs, the antitumor efficacy of the DLE mutant mAb (DLE-TGT) was also assessed. Unlike SEA-TGT, DLE-TGT treatment did not induce substantial antitumor activity, tumor growth delays, or curative responses observed with SEA-TGT treatment (**Figures 3H, L**).

### Effect of FcγR co-engagement on Treg depletion *in vitro*

**Figure 4:**
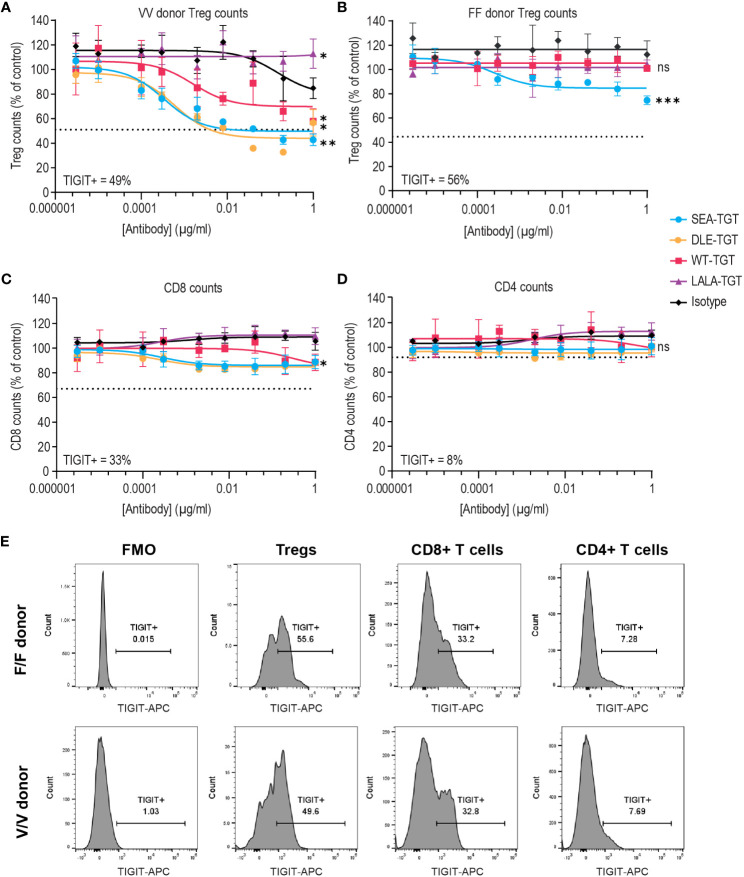
FcγR co-engagement enhances Treg depletion *in vitro*. PBMCs were treated with TIGIT antibodies on different backbones or an hIgG1 isotype control and Treg depletion was assessed in an **(A)** V/V FcγRIIIa allele donor and **(B)** F/F low-affinity FcγRIIIa donor. **(C, D)** CD8+ T cell **(C)** and CD4+ T cell **(D)** depletion was also assessed in the V/V FcγRIIIa allele donor. Numbers on inset represent the percentage TIGIT positive cells at time = 0, dashed lines indicate level equivalent to total TIGIT^+^ cell depletion based on TIGIT levels at time 0. p values that denote statistical significance (* < 0.05, ** < 0.005, ***0.0005, ns denotes no statistical significance) between the isotype group and anti-TIGIT-treated groups were determined using a one-way ANOVA with Dunnett’s multiple comparisons test. **(E)** Mean fluorescence intensity (MFI) of denoting expression level of TIGIT on each cell population in both the F/F and V/V donor is represented via histograms.

To characterize the underlying mechanisms driving the trends for increased antitumor and curative activity of SEA-TGT, *in vitro* evaluation of the different functionalities associated with the effector function-enhanced Fc backbone was performed. The first mechanism investigated was ADCC because TIGIT expression within the tumor bed is primarily on immunosuppressive Tregs and dysfunctional CD8+ T cells (**Figure 1** and **Supplemental Figure 1**). Removal of these cells through ADCC could alleviate negative signals within the tumor microenvironment (TME) and contribute significantly to antitumor activity. To evaluate the effects of the anti-TIGIT mAb backbone on the depletion of TIGIT^+^ cells, human whole peripheral blood mononuclear cells (PBMCs) from a high-affinity FcγRIIIa allele-expressing donor and a low-affinity donor were treated with increasing concentrations of the TIGIT antibodies. Each antibody elicited a different degree of depletion of the Tregs that was correlated to the Fc-engagement status of the backbone (**Figure 4A**). The FcγR inactive LALA-TGT did not induce loss of any T cells in any donors tested (**Figures 4A, B**). All the effector active antibodies, WT, DLE, and SEA, drove statistically significant TIGIT^+^ Treg depletion in the high affinity V/V donor (**Figure 4A**). However, WT-TGT failed to drive significant Treg depletion in a low affinity F/F FcγRIIIa allele donor, while, SEA-TGT treatment resulted in depletion of 25% of total Tregs, a loss of ~45% of the total TIGIT^+^ Tregs in this donor (**Figure 4B**); this depletion was statistically significant at the highest dose used. These data highlight the potential of SEA-TGT to benefit a broader range of patients, independent of their FcγRIIIa genotype.

While depletion of immune suppressive Tregs is beneficial, concomitant loss of functional antitumor CD8^+^ T cells could potentially mitigate antitumor responses. Therefore, loss of either CD8^+^ or CD4^+^ T cells was examined (**Figures 4C, D**). SEA-TGT treatment resulted in only minimal depletion of either cell type, in accordance with the lower expression of TIGIT on these cells (**Figure 4E**). This differential in TIGIT expression between Tregs and CD4 and CD8 T cells is also maintained in the tumor microenvironment, demonstrated by higher levels of 5 different lung cancer dissociated tumor cultures (DTC), vs normal peripheral cells (**Supplemental Figure 1**).

To determine if this Treg depletion phenotype is maintained within a tumor microenvironment the level of CD4, CD8 and Tregs were evaluated in response to SEA-TGT, WT-TGT or LALA-TGT in mouse syngeneic tumors. In a CT26 tumor model, some depletion of total CD4 cells was seen, but it was dependent simply on an intact backbone as there was no large difference between WT-TGT or SEA-TGT intratumoral cell levels and no changes were seen with LALA (**Supplemental Figure 2A**). A more Fc-tuned effect was seen for CD8 T cell levels in the tumor, where no change was seen with LALA but there was incremental increases in response to WT-TGT and the Fc-enhanced SEA-TGT (**Supplemental Figure 2B**). A similar Fc-tuned decrease in Tregs was also observed though because of more intra-tumor heterogeneity, this was less prominent and not statistically significant (**Supplemental Figure 2C**). The tumor modulating effect of SEA-TGT was examined in another syngeneic model, MC38, and here a more prominent, significant decrease of Tregs was observed (**Supplemental Figure 2E**), though no changes in CD4 or CD8 were seen in this model. These data suggest that the *in vitro* human changes can be recapitulated *in vivo*, though are likely model and baseline tumor microenvironment dependent in terms of magnitude.

### Activation of APCs by anti-TIGIT mAbs

**Figure 5:**
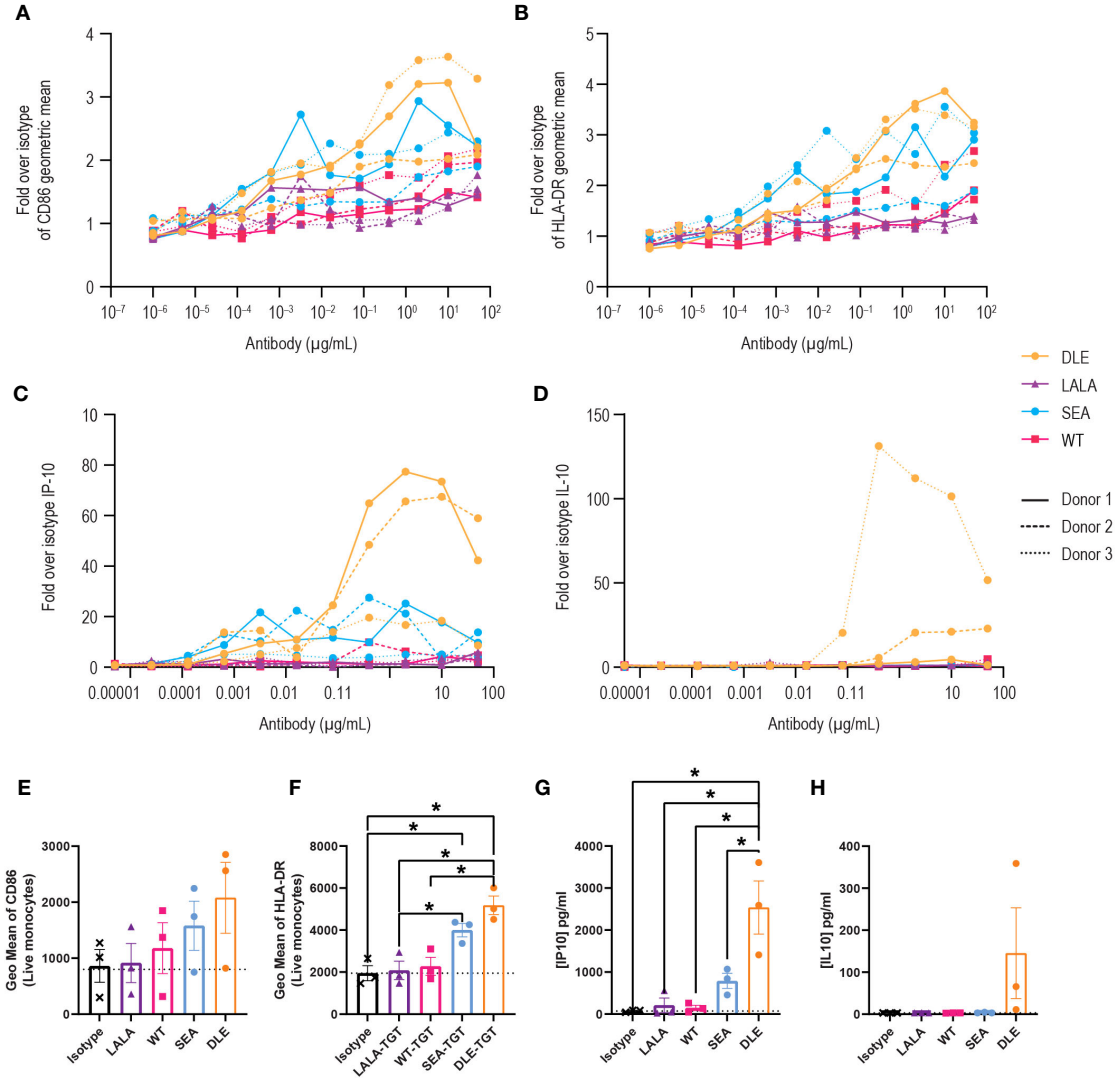
Activation of antigen-presenting cells by anti-TIGIT mAbs is reliant on intact FcγR interactions and is greatly enhanced with a nonfucosylated backbone. PBMCs were stimulated with the different concentrations of the indicated anti-TIGIT and assessed for CD86 **(A)** or HLA-DR **(B)** levels on CD14+ cells 24 hrs later. Data are depicted as the fold increase in geometric mean for the indicated marker over isotype alone in response to a dose range in three separate donors. Supernatants from cultures were also harvested and analyzed for cytokine production using a multiplex kit. Data are graphed for IP-10 **(C)** and IL-10 **(D)**. Summary data at a saturating concentration of 2 µg/ml are denoted for CD86 **(E)**, HLA-DR **(F)**, IP-10 **(G)** and IL-10 **(H)**. p values that denote statistical significance (* < 0.05) were determined using a one-way ANOVA with Dunnett’s multiple comparisons test; groups that did not reach statistical significance are not denoted.

To investigate how FcγR binding of anti-TIGIT mAbs to innate cells might account for the enhanced activity of SEA-TGT (relative to WT-TGT and DLE-TGT), we probed APC activation *in vitro*. PBMCs were stimulated with anti-TIGIT antibodies with various backbones, followed by assessment of activation through evaluation of surface activation markers (CD86 and HLA-DR) and inflammatory cytokine production. Treatment with Fc-intact antibodies increased not only the levels but also the total percentages of cells positive for the co-activation markers CD86 (**Figures 5A, E**) and human leukocyte antigen HLA-DR (**Figures 5B, F**). Greater activation of innate cells visualized by surface co-receptor expression was enhanced over isotype or LALA-TGT when SEA-TGT was used. DLE-TGT, which also increased FcγR binding, drove similar levels of surface co-receptor up-regulation. Because of high donor to donor variability, likely as a result of varying numbers of innate immune cells, statistical significance between groups was not seen with CD86 levels but was noted for HLA-DR levels.

APC activation of T cells requires not only MHC-presented antigens and co-stimulation, but a third signal that comes from an appropriate milieu of cytokines. We investigated how anti-TIGIT antibodies impact the type and level of cytokines produced by innate human cells. Generally, WT-TGT treatment resulted in minimal cytokine responses. However, interferon gamma-induced protein (IP-10), which is important for antigen-dependent T cell responses, increased in a dose-dependent manner, but only with the Fc-enhanced SEA-TGT and DLE-TGT mAbs (**Figures 5C, G**). While the maximal levels of this responsive cytokine were higher with DLE-TGT, the EC_50_ was lower, meaning the nonfucosylated SEA-TGT antibody exhibited higher potency to induce this cytokine. Importantly, a delineation between these antibody backbones was seen when the levels of inhibitory cytokine IL-10 were measured; only DLE-TGT drove any production of this cytokine in the culture system in response to increasing concentrations of antibody (**Figures 5D, H**). The levels of IL-10 induced by the DLE-TGT were highly variable, again likely because of varying levels of innate cells in various donors, and thus were not statistically significantly different than what was seen with SEA-TGT. This selective skewing of SEA-TGT to an interferon response and avoidance of IL-10 generation seen with DLE-TGT which engages the inhibitory FcγRIIb receptor, also correlates with their ability to drive an antitumor response (**Figure 3**).

**Figure 6:**
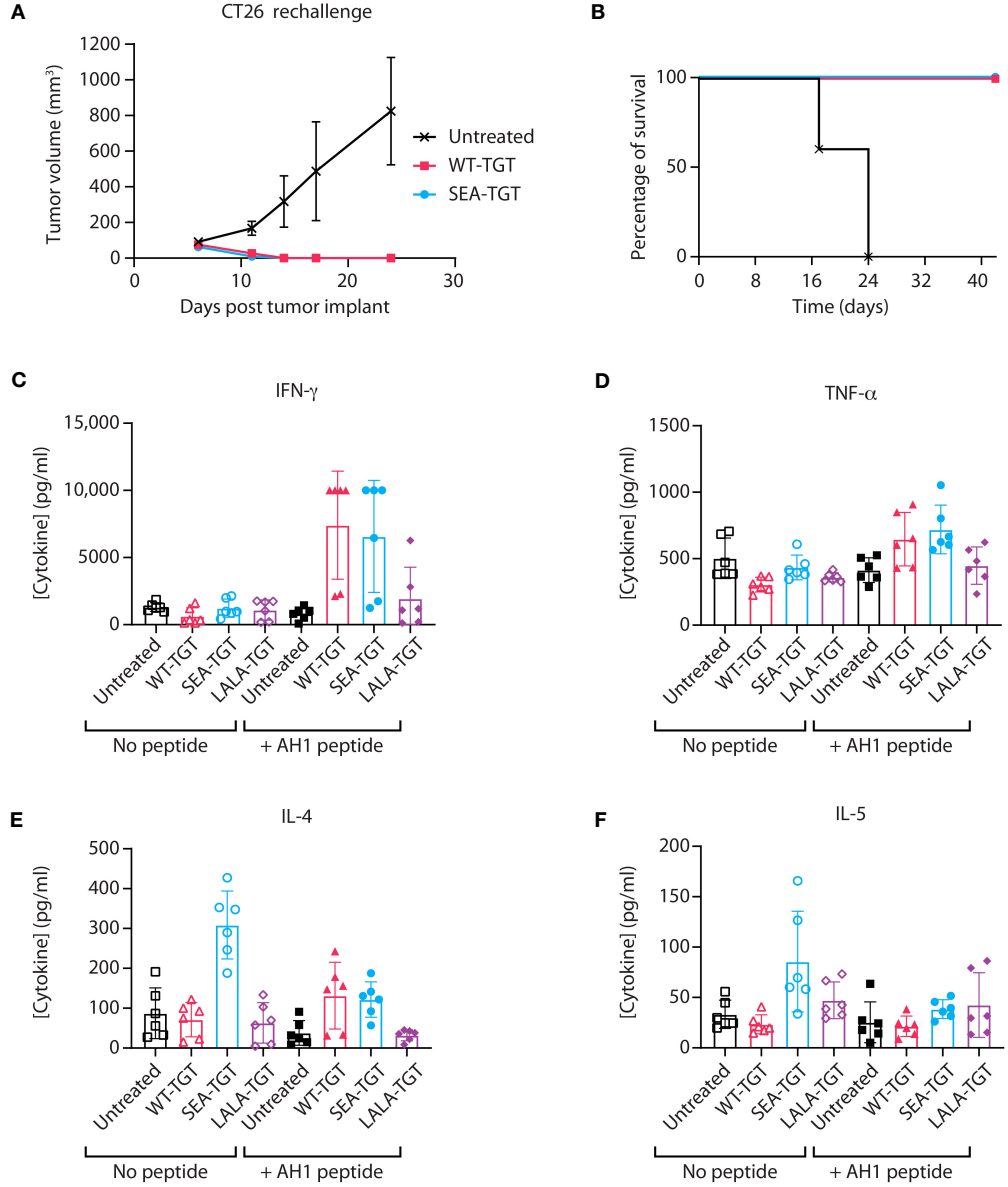
Activation of antigen-specific T cell response by anti-TIGIT antibodies is reliant on intact FcγR interactions. **(A, B)** Balb/c mice that were implanted with CT26 tumors, treated with the indicated anti-TIGIT antibody every 3 days for six doses (Q3dx6) and cured of all tumors, were kept for several weeks post complete regression. CT26 cells were then reimplanted into the previously cured mice and followed for tumor growth over time **(A)** and survival **(B)**. **(C–F)** When mice that were implanted with CT26 tumors reached 100 mm^3^, the animals were treated with the indicated anti-TIGIT mAb at 1 mg/kg every 3 days for six doses (Q3D×6). At 24 hours after the sixth dose, spleens were harvested from animals and splenocytes were left unstimulated (“No peptide”) or restimulated with the AH1 peptide for 72 hours (“+ AH1 peptide”); this was followed by analysis of supernatants from cultures for cytokine production using a multiplex kit for IFN-γ **(C)**, TNF-α **(D)**, IL-4 **(E)**, and IL-5 **(F)**.

Having observed SEA-TGT’s activation of APCs, we hypothesized that SEA-TGT could enhance tumor-specific CD8^+^ T cell responses and examined the generation of these responses using a tumor rechallenge model. Animals that had previously shown curative antitumor (CT26) responses to anti-TIGIT mAb treatment were rechallenged with tumors 9 weeks after initial implantation and 5 weeks post cure. 100% the of animals that had originally been cured were able to fully reject the subsequent tumor challenge (**Figures 6A, B**), highlighting the generation of long-lasting curative antigen-specific CD8^+^ T cells following anti-TIGIT mAb treatment. This protective immunity was seen in animals treated with either WT-TGT or the Fc-enhanced SEA-TGT; both treatments resulted in curative responses upon rechallenge, although more initial curative responses were seen with SEA-TGT treatment. 

To further investigate how these different anti-TIGIT mAbs affect antigen-specific T cell responses against tumors, we utilized an *ex vivo* antigen recall system. Mice implanted with established CT26 tumors were treated with the various anti-TIGIT mAbs. To analyze generation of antitumor T cell responses, spleens from mice were harvested and restimulated with the predominant CT26 CD8^+^ T cell peptide AH1 (27, 28) for 72 hours, followed by analysis of cytokine induction. In response to restimulation with the tumor-specific peptide, induction of the CD8+ T cell effector cytokine interferon gamma (IFN-γ) was greatly enhanced from splenocytes of CT26-bearing mice treated with an anti-TIGIT antibody with an intact Fc backbone (**Figure 6C**). Induction of tumor necrosis factor alpha (TNF-α), IL-4, or IL-5 in response to restimulation was not greatly enhanced (**Figures 6D–F**). Interestingly, in unstimulated cultures, splenocytes from animals treated with SEA-TGT showed higher levels of IL-4 and IL-5 at baseline, possibly suggesting there were still residual CD4+ T cell effects of treatment (**Figures 6E, F**). Importantly, the presence of protective immunity upon rechallenge and evidence for antigen-specific CD8^+^ T cell responses provides evidence that SEA-TGT does not deplete functional antitumor cytotoxic T cells, despite possessing enhanced effector function.

### Influence of underlying immune microenvironments on the responsiveness of SEA-TGT treatment

**Figure 7:**
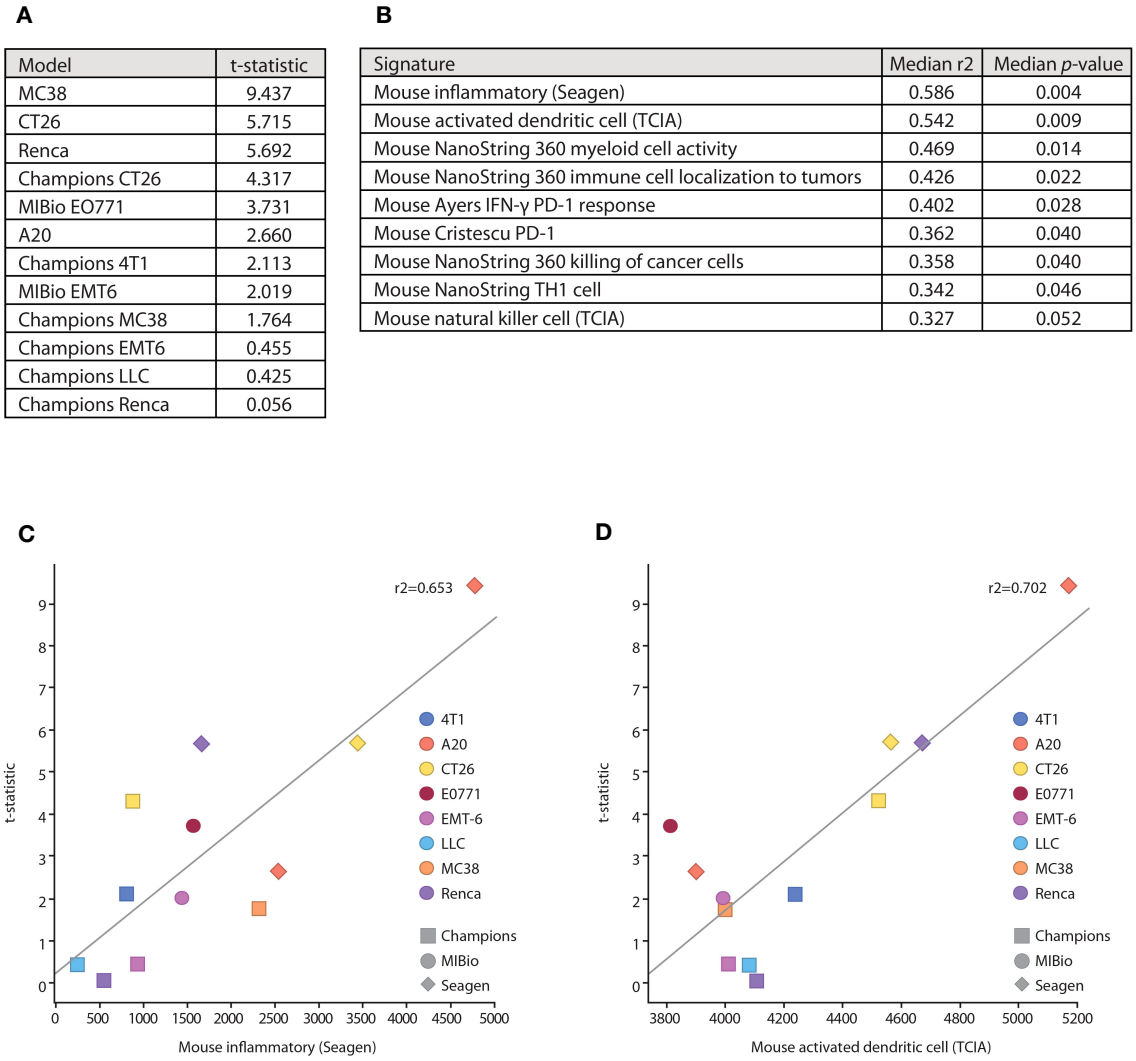
Underlying immune microenvironments correlate with responsiveness to SEA-TGT treatment. **(A)** Response to SEA-TGT treatment at 1 mg/kg across various syngeneic models are denoted using a t statistic metric, where larger numbers denote more significant differences from untreated tumors (refer to Materials and Methods). **(B)** Baseline tumor microenvironment gene signatures most significantly correlated with response to SEA-TGT, with the corresponding median R2 and *p*-values calculated across six different gene signature scoring methods. **(C, D)** Example gene signature and treatment response correlation plots using the ssGSEA gene signature scoring method, for the murine inflammatory signature **(C)** and activated dendritic cell signature **(D)**. The x-axis is the signature score using ssGSEA, and the y-axis is the treatment response t-statistic. Points are colored by syngeneic tumor type, while the point shape denotes the institution where the model was run.

To determine how these *in vitro* mechanisms of action that are enhanced by SEA-TGT correlate to *in vivo* responses and to get a deeper understanding of the TMEs and tumor types that would be responsive to SEA-TGT treatment, the antitumor activity of SEA-TGT was evaluated across disparate syngeneic model systems. The activity of SEA-TGT in these models was quantified by evaluating the average tumor volume in the mice of each treated and untreated group. The significance of the response to SEA-TGT in each model was determined using a t-statistic comparing average tumor volumes between the treated and untreated groups, with higher numbers denoting greater responsiveness (**Figure 7A**). SEA-TGT showed broad and generally strong activity across models, with a select set of tumors showing modest activity.

To understand how the underlying base immune TMEs might impact the responsiveness to SEA-TGT treatment, we correlated baseline signatures of immune repertoires in each of these systems to treatment response. Untreated tumors from each model, some run at different institutions, were profiled using RNA-seq. A total of 74 unique immune-related gene signatures (obtained from a number of sources, including a majority from NanoString and The Cancer Immunome Atlas [TCIA]) were scored against each model using RNA-seq quantification. Scores for each signature were then correlated with treatment response across all models (**Figures 7B–D**). The gene signatures most highly correlated with response to SEA-TGT included a general inflammatory score, APCs (myeloid cell activity, activated dendritic cells), NK cells, and signatures associated with T cell responses (Th1 cells, tumor cell killing) (**Figure 7B**). All of these signatures support the corresponding *in vitro* mechanisms that were utilized by Fc-intact anti-TIGIT mAbs (i.e., NK cell-mediated Treg depletion) as well as those unique to Fc-enhanced SEA-TGT (i.e., the activation of APCs and induction of antigen-specific CD8^+^ T cells). These data support the unique mechanism of the nonfucosylated anti-TIGIT therapy and define underlying immune microenvironments that are predisposed to be more responsive to anti-TIGIT treatment.

Interestingly, two of the other highly correlated gene signatures associated with response to anti-TIGIT treatment include signatures predicting responses to PD-1 therapy (**Figure 7B**). These signatures were developed from baseline biopsies of human patients treated with anti-PD1 therapy, with responders demonstrating IFN-γ-enriched microenvironments at baseline (29, 30). The parallels between underlying anti-TIGIT responsiveness and a PD-1 responsive environment suggested potential synergy between the two agents.

### Enhancement of existing tumor treatments by anti-TIGIT mAbs

**Figure 8:**
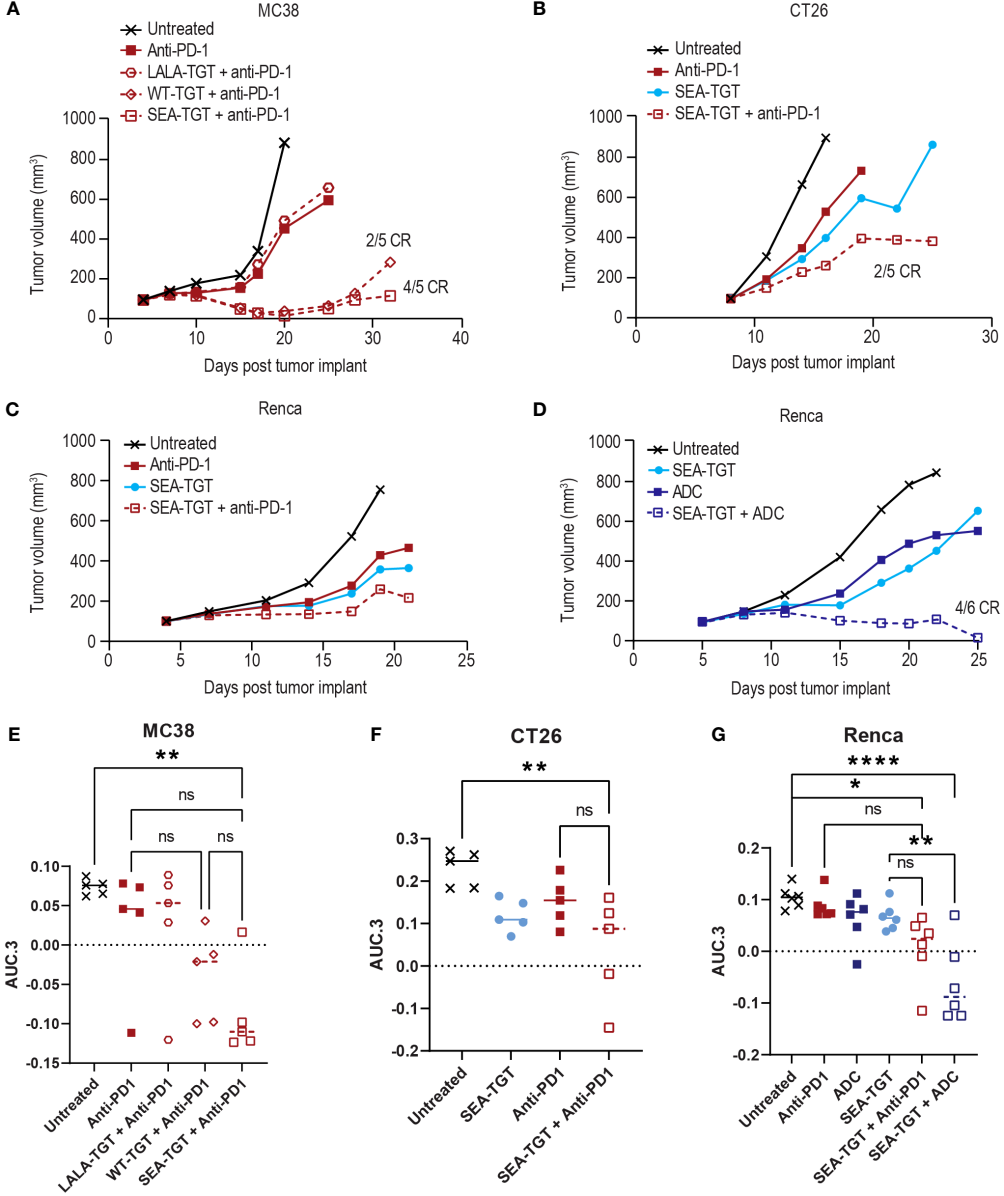
Anti-TIGIT antibodies can drive enhanced activity of anti-PD-1 treatment in syngeneic models but the combinatorial activity is also dependent upon FcγR co-engagement of the TIGIT molecule. Graphs show tumor growth curves in MC38 colon, CT26 colon, or Renca renal cell carcinoma models in mice. MC38-bearing **(A)**, CT26-bearing **(B)**, or Renca-bearing **(C)** animals (5, 5, and 6 animals per group respectively) were dosed with indicated anti-TIGIT treatment or anti-PD-1 antibody at 0.1 mg/kg every 3 days for three doses when tumors reached 100 mm^3^. Tumor growth was followed over time and complete responses (CR) in each group are denoted on the graph. Renca-bearing animals **(D)** were also treated with a single dose of a tumor-targeted MMAE ADC at 1 mg/kg in combination with SEA-TGT at 0.1 mg/kg every 3 days for three doses (Q3D×3) and followed for growth over time. **(E-G)** Overall antitumor activity of the treatment arms across different syngeneic models. Antitumor activity is represented as normalized area under the tumor-response curve (AUC.3) per Guo et al. (BMC Cancer 19, 718 (2019). Tukey’s Honestly Significant Difference adjusted p-values for differences in AUC.3 for each treatment arm, represented as individual animal responses, were run and are denoted. ns, not significant, p values denoted are * < 0.05, ** < 0.005, **** < 0.0001, ns denotes not statistically significant.

To determine whether blockade of an additional immune checkpoint could further boost the responsiveness to anti-TIGIT treatment, we evaluated the antitumor activity of anti-PD-1 treatment in combination with the anti-TGT mAb variants in several models. Addition of the Fc-muted LALA-TGT to a suboptimal dose of an anti-PD-1 treatment was not sufficient to enhance its antitumor activity (**Figures 8A, E**). This finding was consistent with results from the use of LALA-TGT as a single agent (**Figures 3E–G**). These data suggest that simple blockade of both checkpoints is not sufficient to drive antitumor activity alone or in combination with other agents. However, when the Fc-intact (WT-TGT) or Fc-enhanced (SEA-TGT) mAbs were combined with the anti-PD-1 treatment, substantial increases in tumor growth delay were observed (**Figures 8A, E**). This enhancement of response to anti-PD-1 treatment by SEA-TGT was also observed in other models, including CT26 and Renca (**Figures 8B, C, F, G**), demonstrating the potential synergy of these immune checkpoint blockade therapies.

The unique activation of APCs (**Figure 5**) and the determination that response to SEA-TGT was correlated with underlying APCs in the TME (**Figure 7B**) also suggest that anti-TIGIT treatment might synergize well with other therapeutic modalities. Specifically, certain chemotherapies, including monomethyl auristatin E (MMAE) (31, 32), known to induce immunogenic cell death, feed into the generation of new anti-tumor T cell responses and further stimulate the immune response. To determine whether SEA-TGT also synergizes with this mechanism of action, Renca tumors were treated with subtherapeutic levels of single-agent MMAE-containing ADC, SEA-TGT, or a combination of both. Monotherapy of either treatment was able to delay growth while combination therapy greatly and statistically significantly enhanced antitumor activity (**Figures 8D, G**).

## Discussion

In this study, we generated data highlighting that TIGIT targeting goes beyond simple removal of a checkpoint blockade but requires secondary signals generated from the Fc backbone that are critical for antitumor activity. Amplification of FcγR signals through preferential binding to activating FcγRs, which is achieved by a nonfucosylated backbone, drives superior curative antitumor activity. To evaluate the mechanism of action of an anti-TIGIT therapeutic, several anti-TIGIT antibodies were identified with demonstrable binding to human, murine, and non-human primate TIGIT, which blocked the interaction between TIGIT and its ligands, CD155 and CD112. From this antibody pool, one antibody clone was selected for further evaluation of its antitumor activity. The variable domain of this lead antibody was cloned onto various human and murine antibody Fc backbones that exhibited differential binding to various FcγRs. We demonstrated that the blocking ability of anti-TIGIT antibodies alone does not substantially contribute to antitumor activity and that FcγR engagement is critical for this activity, likely through activation of APCs. Further, the presence of a nonfucosylated Fc backbone in the anti-TIGIT antibody resulted in even greater immune responses and antitumor activity, compared with antibodies containing a WT or otherwise modified Fc backbone.

One of the key findings of this study was that TIGIT checkpoint blockade alone was insufficient for antitumor activity, which contrasts with some of the initial observations that TIGIT has T cell-intrinsic effects and that TIGIT signaling directly inhibits T cell activation (7), but has been subsequently supported by several publications (14). Direct T cell inhibition through TIGIT’s ITIM domain, and its similarity to other IgG superfamily members such as PD-1, suggested that blocking TIGIT in TMEs could relieve an inhibitory signal to reinvigorate exhausted tumor-specific memory T cells. However, the inability of the Fc-muted mIgG1 backbone and LALA inactive mIgG2A anti-TIGIT mAbs to drive meaningful antitumor activity across multiple syngeneic models demonstrate this mechanism alone is not sufficient. Instead, depleting these TIGIT-positive, suppressive Tregs and terminally exhausted T cells may produce a more profound therapeutic effect. Exhausted progenitors, marked by TCF1/TCF7 expression (33, 34), are thought to act as a reservoir of T cells with high proliferative capacity that can continuously regenerate short-lived exhausted effector T cells, and thus would likely benefit the most from reactivation. However, fully terminally exhausted cells (marked by high TIM-3 expression (35)), have been described as unresponsive to checkpoint blockade immunotherapy (36). In the NSCLC dataset from Guo et al. (18), and in our own internal NSCLC dataset, levels of TIGIT among cytotoxic T cells were highest in terminally exhausted T cells within the tumor bed, which supports the notion that relief of the TIGIT checkpoint alone through antibody-mediated blockade would likely be insufficient to drive antitumor responses, given that these cells lack any further reactivation potential. Thus, it is likely that to target TIGIT most effectively in the TME, the mechanism of action will need to leverage the Fc region of the antibody to trigger their activation of FcγR expressing innate cells to drive removal of the inhibitory TIGIT^+^ cells. Results from subsequent studies also support our finding that the Fc backbone has a key role to play in driving antitumor activity (14, 37). The findings for anti-TIGIT mAbs contrast with those for other checkpoint molecules such as PD-1, TIM-3, and LAG3, as inhibitors of these checkpoints that retain intact Fc effector function may result in compromised antitumor activity (38–40).

We further characterized the role of the anti-TIGIT mAb’s Fc backbone in immune activation by modifying the backbone to reduce or enhance FcγR engagement (relative to the WT anti-TIGIT mAb, WT-TGT), generating Fc-muted (LALA-TGT) or Fc-enhanced (SEA-TGT and DLE-TGT) anti-TIGIT antibodies. Interestingly, treatment with the Fc-enhanced SEA-TGT resulted in more complete responses and increased delay of tumor growth relative to WT-TGT. While the engagement of the mIgG2a backbones with murine FcgRs approximates human binding, there are known differences in FcgR expression between species which could confound translation of findings to human.

We next sought to elucidate the mechanism by which SEA-TGT drives the enhanced antitumor activity. One of the main Fc-mediated mechanisms of action of therapeutic antibodies is ADCC to drive removal of antigen-expressing cells through NK cell bridging and activation. Compared with WT-TGT, SEA-TGT induced a more complete depletion of TIGIT^+^ Tregs. Notably, very similar depletion was seen between the DLE mutant Fc backbone and the SEA Fc backbone. Although SEA-TGT and DLE-TGT both showed enhanced binding to the activating FcγRIIIa receptors, SEA-TGT showed decreased/low binding to the inhibitory FcγRIIb receptor, demonstrating a skewed Fc interaction profile (**Figures 3A–D**). These data demonstrate that the balance between activating and inhibitory FcγR engagement does not contribute to ADCC induction activity and coupled with the finding that there were differences in antitumor activity based on FcγR bias (**Figure 3H**), suggest that although immunosuppressive Treg depletion likely plays a role in the antitumor response to TIGIT therapy, there are other mechanisms of activity more efficiently exploited by the SEA Fc backbone.

To characterize other potential mechanisms that may contribute to the observed antitumor activity, we investigated APC activation in response to anti-TIGIT treatment. FcγRs are expressed on a wide array of innate immune cells and their engagement can also function to generally activate these cells through an intracellular ITAM domain. Conversely, the inhibitory FcγRIIb receptor contains an ITIM domain that, when triggered, signals through an SH2 domain to serve as a negative feedback loop to inhibit antigen-uptake cellular activation. Thus, activation of APCs can occur upon antibody Fc engagement of FcγR expressed on innate immune cells but can also be shut off when the inhibitory FcγRIIb is engaged (41). Furthermore, the interplay between TIGIT and APCs has been established previously as TIGIT was originally described as indirectly inhibiting T cell responses through modulation of APC function (1). The fact that both SEA-TGT and DLE-TGT resulted in similar levels of APC surface co-receptor expression, suggests that engagement of FcγRIIIa and FcγRIIb can drive similar levels of surface APC activation. However, in contrast to SEA-TGT, treatment with DLE-TGT resulted in concomitant large increases in the inhibitory cytokine IL-10 production, reflecting the differing affinity of these mAbs for the inhibitory Fc receptor. These data suggest that selective enhanced engagement of the FcγRIIIa receptor by the nonfucosylated SEA-TGT optimally activates APCs in a manner that can maximize antigen+ CD8^+^ T cell generation, underscoring the central role of APC activation in the antitumor activity of anti-TIGIT therapy. While enhanced binding to all FcγRs can undoubtedly activate APCs to drive short-term antitumor activity, it is likely that the concomitant induction of inhibitory feedback mechanisms results in sub-par long-term therapeutic T cell responses and curative antitumor responses, as has been previously reported (42). Long-term protective immune response against tumors requires presentation of antigens by activated APCs to drive optimal activation of tumor-specific memory T cells. Activation of the APCs in concert with CD8^+^ T cell-activating cytokines, such as type I interferons, suggests that the nonfucosylated SEA-TGT should be superior at driving generation of these cells.

A key strength of this study is its detailed characterization of the Fc backbone, which included biophysical, *in vitro*, and *in vivo* assessments. Another strength is the fact that the antitumor activity of the mAbs was assessed in multiple syngeneic model systems and across several institutions, increasing the generalizability of the findings and confirming that *in vitro* mechanisms of action correlate to responsiveness *in vivo*. Our findings helped identify a novel modality for this class of antibodies, prompting us to reconsider the definition of a checkpoint and how TIGIT might function in immune evasion and tumor progression. One possible weakness of the study is that preclinical studies and clinical translation of these findings will not be fully realized as clinical data for the TIGIT mAbs with different Fc backbones. To evaluate the clinical efficacy of the SEA-TGT antibody, a phase I, open-label, multicenter, dose-escalation/expansion study (43) (NCT04254107) was recently initiated; study recruitment is ongoing, targeting patients with either solid cancers or lymphomas.

In conclusion, we show that specific characteristics of the Fc backbone of the anti-TIGIT antibody are critical for antitumor activity preclinically across several syngeneic murine tumor models. This activity is likely due to a combination of multiple mechanisms of action including APC activation, T cell priming, and NK-mediated depletion of APCs; these cells included suppressive T regulatory cells and terminally exhausted T cells, which were found to be higher expressers of TIGIT in primary human lung tumors. We maximally capitalized and specifically enhanced these multiple modes of action through the use of a nonfucosylated backbone to create the SEA-TGT mAb, which was able to drive enhanced preclinical antitumor activity.

## Materials and methods

### Experimental design

The main objective of this study was to characterize the determinants of the antitumor activity demonstrated by anti-TIGIT antibodies. To achieve this, we generated a series of anti-TIGIT mAbs that bound with high affinity to human, non-human primate, and murine TIGIT. The lead mAb clone was selected for further characterization of the antibody backbone, through the generation of variants with differing Fc regions. The antitumor activity of the Fc variants was tested in several syngeneic murine models, with age and sex (female) matched mice randomized (to give equivalent mean baseline tumor sizes in each group) into treatment groups. PMBCs from normal healthy donors were used to characterize depletion of CD8^+^ and CD4^+^ T cells and Tregs as well as APC activation by the anti-TIGIT mAbs. Tumors from murine models were profiled using RNA-seq followed by scoring of the profiled samples against immune-related gene signatures.

### Generation of anti-TIGIT mAbs

Anti-TIGIT mAbs were generated by screening eight naïve human synthetic yeast libraries to identify high-affinity antibodies (44) that bound human, non-human primate, and murine TIGIT. Primary screening comprised four panning steps: positive selection using biotinylated monovalent human antigen and flow cytometry to detect binding; a negative selection round; a TIGIT antigen titration round; and cross-species binding evaluation to non-human primate and murine TIGIT via flow cytometry. Selection rounds were repeated until a population with the desired receptor binding and checkpoint blockade characteristics was obtained. After the final round of sorting, yeast were plated and individual colonies were picked for characterization. In total, 728 clones were sequenced, yielding 350 unique heavy chain/light chain combinations. From these unique clones, 65 were selected for production and further evaluation, representing 12 heavy chain variable germlines and nine light chain variable germlines.

### Anti-TIGIT antibody production

Fc variants used with the lead anti-TIGIT mAb clone included wild-type (WT), L234A/L235A/P329G (LALA), and S239D/A330L/I332E (DLE) human IgG1, as well as murine IgG1, IgG2a, or IgG2a LALA (numbering according to EU nomenclature) Fc backbones. The cloning, expression, and purification of the mAb clones and subsequent binding experiments are described in detail in the Supplementary Materials and Methods.

### Blockade of CD155 and TIGIT interaction

hCD155-Fc (Sino Biological 10109-H02H) and mCD155-Fc (Sino Biological 50259-M41H) were conjugated to Alexa Fluor 647 (Thermo Fisher). Cells (2 × 10^5^) expressing 293-hTIGIT or 293-mTIGIT were co-incubated with 1 μg/ml CD155-Fc-Alexa Fluor 647 and a 12-point, two-fold titration (10–0.005 μg/ml) of each anti-TIGIT antibody or an isotype control antibody. Samples were analyzed on a CytoFLEX flow cytometer (Beckman Coulter). Median fluorescence intensity (MFI) of the forward scatter/side scatter gated population was determined for each antibody concentration. Percentage blockade was calculated relative to the MFI of the no anti-TIGIT antibody control. Nonlinear regression of Log(X) transformed data was performed in GraphPad Prism 6.

### Antitumor activity of TIGIT

Female Balb/c or C57BL/6 mice (Envigo), 6–8 weeks of age, were subcutaneously implanted with tumor cells on the flank on day 0. The tumor cell lines implanted into Balb/c mice were CT26 (1 × 10^5^ cells), Renca (2 × 10^6^ cells), A20 (5 × 10^6^ cells), EMT6 (1 × 10^6^ cells at MI Bioresearch), and E0771 (5 × 10^5^ cells at MI Bioresearch). MC38 (1 × 10^6^ cells) were implanted into C57Bl/6 mice. Tumor cell lines were obtained from ATCC. When mean tumor size reached 100 mm^3^ (measured with calipers: volume [mm^3^] = 0.5 × length × width^2^, where length is the longer dimension), mice were randomized into treatment groups. The animals were treated with anti-TIGIT antibodies (0.1 or 1 mg/kg) every 3 days for three to six doses intraperitoneally. Tumor size was followed over time. For rechallenge experiments, animals that were fully cured of tumors during the initial course of treatment were allowed to recover for several months. CT26 cells were then implanted on the opposite flank to initial implantation. These animals were followed for tumor growth over time.

For all experiments, the average tumor volume was calculated for each animal as the area under the curve of tumor volume versus time, divided by the number of days on study. Efficacy was gauged for each model by calculating the t-statistic comparing the average tumor volumes of treated mice versus untreated mice, using the Welch Two Sample *t*-test under the alternative hypothesis that the true difference in means between the two groups is not equal to 0 (R function *t* test). Antitumor efficacy was also assessed as part of a SynScreen at Champions Oncology in MC38, CT26, LLC, 4T1, EMT6, B16F10, and Renca tumor models per the contract research organization’s standard operating procedure.

For combination studies, a surrogate murine cross-reactive anti-PD-1 antibody was used (clone 29F1.A12, InVivoPlus; Bio X Cell) and dosed at 0.1 mg/kg every 3 days for three doses (Q3D×3) when tumors reached 100 mm^3^. For ADC combination studies, an EphA2 tumor-targeted antibody on a murine IgG2a backbone was conjugated with MMAE at an average drug–antibody ratio of 4 and dosed once at 1 mg/kg.

### Human tumor profiling by scRNA-seq

Publicly available data from scRNA sequencing of NSCLC T cells (18) (http://lung.cancer-pku.cn) were leveraged to assess the expression levels of TIGIT across intratumoral T cell subpopulations. T cells originating from peripheral blood or normal tissues were excluded from the analysis. For internally generated scRNA-seq data, dissociated primary human lung tumors (DTCs) from three patients were obtained from Discovery Life Sciences (DLS), who isolated T cells and performed scRNA sequencing and data analysis using the methodology described in the Supplementary Materials and Methods.

### FcγR cell binding

Anti-TIGIT mAbs were tested for FcγR engagement in Chinese hamster ovary (CHO) cell lines expressing different FcγRs (45). Cells were incubated with WT-TGT, SEA-TGT, LALA-TGT, isotype, and SEA isotype at 4°C for 1 hour. Cells were then washed and stained with antihuman IgG Fc antibody in Becton Dickinson stain buffer and incubated for 30 minutes at 4°C. Cells were washed in stain buffer and resuspended in phosphate buffered saline (PBS) +1% paraformaldehyde. Cells were then analyzed using an Attune NxT flow cytometer (Thermo Fisher) and graphed by geometric MFI.

Anti-TIGIT mAb binding kinetics with human FcγRI, FcγRIIa H131, FcγRIIa R131, FcγRIIb, FcγRIIIa F158, FcγRIIIa V158 were assessed by BLI (biolayer interferometry). Biotinylated recombinant human Fc-receptor proteins were diluted in immobilizing buffer (0.1% BSA, 0.02% Tween20, 1x PBS pH 7.4) and loaded onto SAX (streptavidin) biosensors (Sartorius) with optimized conditions. After an initial baseline in immobilizing buffer to ensure the Fc-Receptors were not dissociating from the biosensors, a second baseline was done in kinetic buffer (1% casein, 0.2% Tween20, 1x PBS pH 7.4 for FcγRI/IIa/IIb/IIIa). Then, serial dilutions of test articles in kinetic buffer were allowed to associate with human Fc-receptors immobilized on biosensors until the top concentration of test articles reached equilibrium with recombinant protein. Lastly, biosensors were incubated in kinetic buffer to allow for antibody dissociation to occur. Sensorgrams capturing the association and dissociation of test articles from human Fc-receptors were generated at 30°C on an Octet HTX system (ForteBio). Reference biosensors with immobilized human Fc-receptors were measured in the absence of test article. Negative control biosensors without immobilized human Fc-receptors were assessed with test articles present at 20 μM to verify the absence of nonspecific binding of the test articles to the SAX biosensors themselves. Data for BLI kinetic experiments were processed and analyzed on the Data Analysis HT software (Sartorius). All sensorgrams were processed with a Y-axis alignment to an average of the last 5 seconds of the second baseline and an inter-step correction aligned to the dissociation step before analysis. The binding kinetic rate constants were calculated by globally fitting the sensorgrams with a 1:1 Langmuir adsorption isotherm model (Rmax unlinked) after a reference subtraction of the human Fc-receptor-loaded sensors in absence of test article.

### Restoration of CD226 signaling

TIGIT effector cells and CD155 CHO cells (Promega) were thawed and cultured. A dose series of antibody was prepared, starting at 200 ng/ml, and this was diluted eight times using a six-fold dilution factor; these dilutions were then plated into white, flat-bottom plates. Dilutions of TIGIT effector cells and CD155 CHO cells were added to the plates prior to incubation for 6 hours at 37°C and 5% CO_2_. The plates were removed and cooled on wire racks in a biosafety cabinet for 20 minutes. To visualize the luciferase signal, Bio-Glo Reagent (Promega) was prepared and added to the plates at a 1:1 ratio per well and allowed to incubate for 15 minutes at room temperature. The plates were then imaged on an EnVision plate reader (Perkin Elmer) and four-parameter logistic curves were obtained through analysis using SoftMax Pro software.

### TIGIT expression levels

Cryopreserved PBMCs from normal healthy donors (purchased from Astarte and Folio Conversant) were thawed, washed in RPMI 1640, then washed with PBS, stained for viability with Zombie Aqua (BioLegend) for 15 minutes at RT, washed in cell staining buffer (PBS containing 2% FBS and 0.05% sodium azide from Gibco and VWR Chemicals, respectively), and Fc-blocked with 5% Human TruStain Fcx (BioLegend) in cell staining buffer for 10 minutes. The cells were then incubated with the following fluorescent antibodies from BioLegend at 4°C for 30 minutes: CD3 AF488, CD4 APC/Fire 750, CD25 BV605, CD127 BV711, CD45RA PerCP-Cy5.5, CCR7 BV421 (all at 1:50 dilution), CD8 AF700 (1:100 dilution), and TIGIT APC (eBioscience, 1:20 dilution). Following staining, cells were washed twice and resuspended in cell staining buffer and analyzed on an Attune NxT flow cytometer (Thermo Fisher). Data were analyzed with FlowJo software and graphed using GraphPad Prism.

Cryopreserved dissociated tumors from 5 non-small cell lung cancer patients (purchased from Discovery Life Sciences) and PBMCs from 2 normal healthy donors (purchased from Astarte and Bloodworks NW) were thawed, washed in RPMI 1640, then washed with PBS, stained for viability with Zombie Aqua (BioLegend) for 15 minutes at RT, washed in cell staining buffer (PBS containing 2% FBS and 0.05% sodium azide from Gibco and VWR Chemicals, respectively), and Fc-blocked with 5% Human TruStain Fcx (BioLegend) in cell staining buffer for 10 minutes. The cells were then incubated with the following fluorescent antibodies from BioLegend at 4°C for 30 minutes: CD2 BV605, CD4 APC/Fire 750, TIGIT PE-Cy7, CD45RA PerCP-Cy5.5, CCR7 PE/Dazzle 594 (all at 1:50 dilution) and CD8 AF700 (1:100 dilution). Following cell surface staining, cells were washed twice then fixed, permeabilized, and stained with Foxp3 BV421 (BioLegend, 1:50 dilution) using the True-Nuclear Transcription Factor Buffer Set (BioLegend) according to the manufacturer’s protocol. Cells were then washed and resuspended in cell staining buffer and analyzed on an Attune NxT flow cytometer (Thermo Fisher). Data were analyzed with FlowJo software and graphed using GraphPad Prism.

### TIGIT-mediated T cell depletion

Cryopreserved PBMCs from normal healthy donors (purchased from Astarte and Bloodworks NW) were thawed, washed, counted, and resuspended in RPMI Medium 1640 containing 10% heat inactivated fetal bovine serum (FBS) and supplemented with a 1 × dilution of 100X MEM nonessential amino acids, sodium pyruvate, GlutaMAX-l, and Pen Strep (Gibco). The PBMCs were then incubated in a 96-well U-bottom plate at 2.5 × 10^5^ cells/well in the absence or presence of increasing concentrations of the indicated anti-TIGIT mAb or human IgG1 control antibody at 37°C and 5% CO_2_. After 24 hours, the cells were washed with PBS, stained for viability with Zombie Aqua (BioLegend), washed in cell staining buffer (PBS containing 2% FBS and 0.05% sodium azide from Gibco and VWR Chemicals, respectively), and Fc-blocked with 5% Human TruStain Fcx (BioLegend) in cell staining buffer for 10 minutes. The cells were then incubated with the following fluorescent antibodies from BioLegend at 4°C for 30 minutes: CD3 AF488, CD4 APC/Fire 750, CD25 BV605, CD127 BV711, CD45RA PerCP-Cy5.5, CCR7 BV421 (all at 1:50 dilution), and CD8 AF700 (1:100 dilution). An aliquot of cells at time 0 was also stained in the same manner and included TIGIT APC (eBioscience, 1:20 dilution) to establish baseline TIGIT levels. Following staining, cells were washed twice and resuspended in cell staining buffer and analyzed on an Attune NxT flow cytometer (Thermo Fisher). Data were analyzed with FlowJo software and graphed using GraphPad Prism.

### Immune cell changes in response to anti-TIGIT mAbs *in vivo*

Female Balb/c mice (Envigo), 6–8 weeks of age, were subcutaneously implanted with 1 × 10^5^ CT26 tumor cells on the flank on day 0. When mean tumor size reached 100 mm^3^ mice were randomized into treatment groups. The animals were treated with 1 mg/kg of the indicated anti-TIGIT antibody every 3 days for three doses intraperitoneally. Tumors were harvested from animals 24 h post 3^rd^ dose and processed into single cells by mashing over a100 µm filter and washing with PBS. RBCs were lysed using RBC lysis buffer (Gibco) per the manufacturers protocol followed by 2x washing in PBS. Single cells were resuspended in FACS buffer (BD), stained with antibodies against CD45 (30-F11), CD4 (RM4-5), CD8 (53-6.7), CD25 (PC61), CD127 (A7R34) and live dead and analyzed on a BD Attune flow cytometer and data was analyzed using FlowJo.

C57/BL6 mice were subcutaneously implanted with MC38 tumor cells on the flank on day 0. When mean tumor size reached 100 mm^3^ mice were randomized into treatment groups. The animals were treated with 1 mg/kg of the indicated anti-TIGIT antibody every 3 days for two doses intraperitoneally. Tumors were harvested from animals 48 h post 2^nd^ dose and processed into single cells by mashing over a100 µm filter and washing with PBS. Single cells were resuspended in FACS buffer (BD), stained with antibodies against CD45 (30-F11), CD4 (RM4-5), CD8 (53-6.7), CD25 (7G7B6), and live dead, followed by fixation with fix/perm buffer and intracellular staining with FOXP3 (FJK-16s) overnight, then washed, resuspended in FACS staining buffer and analyzed on a BD Attune flow cytometer and data was analyzed using FlowJo.

### TIGIT-mediated APC activation

Cryopreserved PBMCs from normal healthy donors were thawed, washed, counted, and resuspended in RPMI 1640 containing 10% FBS. The cells were then incubated in a 96-well U-bottom plate at 2 × 10^5^ cells/well in the absence or presence of increasing concentrations of the indicated anti-TIGIT mAb or a human IgG1 control antibody at 37°C and 5% CO_2_. After 24 hours, the cells were spun down and supernatant was harvested. Supernatants were analyzed using a MILLIPLEX multiplex kit (EMD Millipore) containing the analytes tumor necrosis factor alpha (TNF-α), macrophage inflammatory protein 1β (MIP1β), monocyte chemoattractant protein-1 (MCP1), interferon gamma-induced protein 10 (IP10), interleukin (IL)-6, IL-4, IL-2, IL-1β, IL-17, IL-12p40, IL-10, and interferon gamma (IFN-γ). The samples were analyzed on a Luminex MAGPIX instrument (Luminex Corp) using xPONENT software. After the supernatants were removed, the cell pellets were washed, Fc-blocked with 5% Human TruStain Fcx (BioLegend), and incubated with the following fluorescent antibodies on ice for 30 minutes according to manufacturer recommendations: Live dead violet (Life Technologies), CD14 AF488 (eBioscience), HLA-DR PE (eBioscience), and CD86 APC (eBioscience). Cells were then washed and analyzed on an Attune NxT flow cytometer (Thermo Fisher) and data were analyzed with FlowJo software.

### Antigen-specific T cell responses

Balb/c mice were subcutaneously implanted with CT26 cells in the flank as described earlier. When tumors reached 100 mm^3^, the animals were treated with six doses of the indicated anti-TIGIT mAb at 1 mg/kg every 3 days. Twenty-four hours after the sixth dose, spleens were harvested from anesthetized animals, processed over a 100 mm filter and washed with PBS. Red blood cells were lysed using red blood cell lysis buffer (Invitrogen) and cells were resuspended in Dulbecco’s Modified Eagle Medium (with 10% FBS). Splenocytes were plated at 1 × 10^6^ cells/well in a flat bottomed 96-well plate and either left unstimulated or restimulated with 100 µg/mL of the AH1 peptide (AnaSpec) for 72 hours at 37°C and 5% CO_2_. Following incubation, supernatants were harvested and evaluated for cytokine production using a MILLIPLEX multiplex kit (EMD Millipore) containing the analytes TNF-α, IL-4, IL-5, and IFN-γ. The assay was analyzed on a Luminex MAGPIX instrument using xPONENT software.

### Syngeneic tumor profiling by RNA-seq and gene signature analysis

Untreated tumors from all models were profiled using RNA-seq. For in-house (A20, MC38, and Renca) and Champions models (EMT6, CT26, LLC, 4T1, MC38, and Renca), frozen untreated tumor samples were prepared and shipped to GENEWIZ for RNA extraction, RNA library preparation using PolyA selection, and sequencing using 150 bp paired-end reads; results were delivered in FASTQ format. For our in-house CT26 model, tumor samples were preserved as formalin-fixed paraffin-embedded (FFPE) blocks and shipped to DLS for RNA isolation/extraction using the Hudson Alpha Discovery FFPE Tissue Extraction Method, RNA library preparation using rRNA-Reduction, and sequencing using 100 bp paired-end reads; results were delivered in FASTQ format. For MIBio/Covance models E0071 and EMT6, FASTQ files for RNA-seq performed on untreated tumors were provided directly from MIBio/Covance. Analysis of gene signatures and correlations with efficacy were performed as described in the Supplementary Materials and Methods.

## Data availability statement

The datasets presented in this study can be found in online repositories. The names of the repository/repositories and accession number(s) can be found below: https://www.ncbi.nlm.nih.gov/, GSE241212.

## Ethics statement

Ethical approval was not required for the studies on humans in accordance with the local legislation and institutional requirements because only commercially available established cell lines were used. The animal study was approved by The Institutional Animal Care and Use Committee (IACUC) at Seagen. The study was conducted in accordance with the local legislation and institutional requirements.

## Author contributions

AS: Conceptualization, Data curation, Formal Analysis, Investigation, Methodology, Project administration, Supervision, Visualization, Writing – original draft, Writing – review & editing. BT: Investigation, Methodology, Visualization, Writing – review & editing. WZ: Investigation, Methodology, Visualization, Writing – review & editing. BG: Investigation, Methodology, Visualization, Writing – review & editing. SL: Investigation, Methodology, Visualization, Writing – review & editing. GG: Investigation, Methodology, Visualization, Writing – review & editing. RH: Investigation, Methodology, Visualization, Writing – review & editing. SW: Investigation, Methodology, Visualization, Writing – review & editing. AB: Investigation, Methodology, Visualization, Writing – review & editing. SP: Conceptualization, Supervision, Writing – review & editing. SG: Project administration, Supervision, Writing – original draft, Writing – review & editing, Conceptualization.
