# Supplementary material for: Nonfucosylation of an anti-TIGIT antibody enhances FcγR engagement, driving innate immune activation and antitumor activity
Source: Front Immunol. 2023 Nov 1;14:1280986. doi: 10.3389/fimmu.2023.1280986 (PMC10654636; doi:10.3389/fimmu.2023.1280986)
Supplement: Supplementary file 1 [file DataSheet_1.docx]

# *Supplementary Material*

# SUPPLEMENTARY MATERIALS AND METHODS

## Anti-TIGIT antibody cloning, expression, and purification

The sequences for the variable domain of the lead monoclonal antibody (mAb) clone and the crystallizable fragment (Fc) variants were synthesized using nontemplate PCR and cloned into either transient or stable expression vectors. The resultant plasmids were then transformed into *E. coli* and unique colonies were used to inoculate liquid media for overnight growth. Plasmid DNA was isolated and purified from the resulting cultures, and the sequences were verified using Sanger sequencing.

For transient expression, a 1:1 ratio of antibody heavy chain and light chain vectors were diluted into OptiPRO SFM medium with ExpiFectamine Chinese hamster ovary (CHO) transfection reagent (Thermo Fisher). The DNA/transfection reagent was then added to an ExpiCHO-S culture in ExpiCHO Expression medium (Thermo Fisher) and cultured for 9 days. For stable expression, 100 µg of a linearized expression vector containing both heavy and light chain genes were electroporated into CHO-DG44 cells. Highly expressing clones were generated by dihydrofolate reductase selection and limited dilution cloning. The final production clone was used to produce the lead anti-TIGIT mAb in a 14-day fed-batch expression. To generate the nonfucosylated sugar-engineered anti-TIGIT mAb (SEA-TGT), a fucose analog was added to the transient or stable expression culture passages before and during antibody production. The cultures were harvested by centrifugation and 0.2 µm filtration.

GE HiTrap mAb Select SuRe columns (Cytiva) were used for the purification of each antibody. Prior to elution, the resin was washed with phosphate buffered saline (PBS) + 0.1% Triton, PBS + 0.5M NaCl, and PBS. Each antibody was eluted using 25 mM Acetic Acid pH 3 Buffer, then buffer exchanged using a 26/60 HiPrep Desalt column (Cytiva) into PBS. Purified antibodies were analyzed by analytical size exclusion high-performance liquid chromatography (HPLC), analytical hydrophobic interaction chromatography HPLC, and reduced glycosylated and deglycosylated polymeric reversed-phased column mass spectrometry (PLRP-MS; quadrupole time of flight [QToF]). Nonfucosylation of SEA-TGT was verified by reduced PLRP-MS (QToF).

## Anti-TIGIT antibody binding and characterization *Human embryonic kidney cells*

HEK 293 cells (from ATCC) were engineered to stably express high levels of human, mouse, or cynomolgus monkey TIGIT by lentiviral transduction. Approximately 1 × 10^5^ parental HEK 293 cells or HEK 293 cells overexpressing human, mouse, or cynomolgus monkey TIGIT were stained with 100 nM of each anti-TIGIT antibody for 5 minutes at room temperature. Cells were then washed twice with buffer (Becton Dickinson stain buffer) and incubated with antihuman immunoglobulin G (IgG) conjugated to phycoerythrin for 15 minutes on ice. Cells were then washed twice with wash buffer and analyzed by flow cytometry on a fluorescence-activated cell sorting Canto II system (BD Biosciences). Fold-over background was calculated as the median fluorescence intensity (MFI) of the anti-TIGIT clone bound to target-positive cells divided by the MFI of the anti-TIGIT clone bound to target-negative cells.

### **Bio-layer interferometry**

ForteBio affinity measurements were performed on an Octet RED384 as previously described (1)(*46*). Briefly, ForteBio affinity measurements were performed by loading IgGs on-line onto antihuman IgG quantitation sensors. Sensors were equilibrated off-line in assay buffer for 30 minutes and then monitored on-line for 60 seconds for baseline establishment. Sensors with loaded IgGs were exposed to 100 nM antigen (dimeric Fc-fusion antigen or monomeric antigen) for 3 minutes, and afterwards were transferred to assay buffer for 3 minutes for off-rate measurement. All binding and dissociation kinetics were analyzed using the 1:1 binding model.

## Human tumor profiling by single-cell RNA sequencing (scRNA-seq)

Frozen samples of primary human lung tumors underwent dead cell removal and T cell enrichment using the Sony MA900 Cell Sorter with a DAPI live/dead cell discriminator and staining for CD45 and CD3. Following dead cell removal and T cell enrichment (CD45^+^ CD3^+^), cell count and viability were assessed using acridine orange/propidium iodide on a Nexcelom Cellometer and cells were washed twice in PBS + 0.04% bovine serum albumin. Following successful isolation, 15,000 cells per sample were loaded into the 10X Chromium chip using the Chromium Single Cell (v3.1 chemistry) with the intention of targeting 10,000 cells/sample for sequencing. 3′ GEX scRNA-Seq libraries were prepared according to manufacturer recommendations. Libraries were quality controlled on the Agilent TapeStation. Libraries were pooled and sequenced on the NovaSeq 6000 S2 100 flow cell targeting an average depth of 25,000 reads per cell (×10,000 cells/sample) or 250 million reads/sample. FASTQ data files containing raw reads for each of the three samples were generated and analyzed separately by the vendor to generate single-cell feature (gene) counts using the function “cellranger count” from 10x Genomics Cell Ranger 5.0.0 (2)(*47*). A single feature count matrix aggregating across all three samples was generated using “cellranger aggr” from 10x Genomics Cell Ranger 6.0.0. From the resulting matrix, cells that had fewer than 1000 total counts; or more than 25% of their counts going to mitochondrial genes (using a previously curated list (3)(*19*)), or which had zero CD3 expression (zero counts for CD3D, CD3E, and CD3G), were excluded. The reduced matrix was reanalyzed using “cellranger reanalyze”, and the data were loaded into R (4.2.2) using the “Read10X” function from the R package Seurat (4.3.0) (4). The standard workflow was performed following the tutorial provided by the Satija Lab (https://satijalab.org/seurat/articles/pbmc3k_tutorial.htmlhttps://satijalab.org/seurat/articles/pbmc3k_tutorial.html). After further quality control (doublet removal), the workflow was rerun in similar fashion. “RunUMAP” and “FindNeighbors” were run using 30 dimensions. A total of 10 clusters were identified using “res=0.5” in “FindClusters” (Figures 1B, C). The boxplot used TIGIT expression retrieved using the default “data” slot in “FetchData” (Figure 1D). CD8^+^ cells were subset by TIGIT expression (TIGIT+ or TIGIT-). “FindAllMarkers” was run on these two subsets, and the top genes are depicted in the heatmap in Figure 1E.

## Syngeneic tumor profiling by RNA-seq and gene signature analysis

FASTQ files from RNA-seq of all models were uniformly processed in-house using a pipeline consisting of adapter trimming using Cutadapt (5) v1.16; read alignment was performed using STAR (6) v2.5.2b, to the mm10/GRCm38 genome and transcriptome annotated by GENCODE and downloaded from UCSC; and isoform and gene-level transcript quantification was conducted using RSEM (7) v1.2.31. The FFPE samples for the in-house CT26 model were run using RSEM parameters for a TruSeq stranded library (--forward-prob=0). As a normalization step, we performed a rescaling of the gene- and isoform-level quantification TPMs (transcripts per million reads) to exclude nonprotein-coding and mitochondrial genes, a step that we have found to improve the clustering of samples with mixed library preparation methods.

Immune-related gene signatures were collected from multiple sources: the NanoString nCounter PanCancer Immune Profiling Panel (8); the NanoString PanCancer IO 360 Panel (9); TCIA set of immune metagenes (10)(*54*); published gene signatures related to anti-PD-1 therapy (11)(*30*); and an internal ("Seagen") inflammatory gene signature comprised of CCL2, CCL3, CCL4, CXCL10, IFNG, IL6, IL12A, IL12B, IL1B, and TNF. This resulted in a total of 74 unique signatures across all sources. Genes in each signature were replaced by their mouse homolog(s) and the resulting signatures were used to score each profiled sample using six different single-sample scoring methods: the simple sum of TPMs across the genes in the signature; the sum of z-scores (12); CERNO (13)(*56*); PLAGE (14); singscore (15)(*58*); and ssGSEA (16)(*59*). All calculations were performed using R, v4.0.1, including the GSVA (17) package, v1.4.0, for their implementations of ssGSEA and the z-score method, and the singscore package, v1.8, for the singscore method (both packages available from Bioconductor [bioconductor.org]). The number of RNA-seq biological replicates for each institution/model ranged from 1 to 6. In the case of multiple replicates, the signature scores were averaged across the replicates, resulting in a single score per institution/model for each signature and each scoring method.

## Signature correlations with *in vivo* efficacy

Signature scores for all 12 institutions/models were then correlated with the *in vivo* efficacy t-statistics across those models, producing a ranking of signatures by their correlation *p*-values (for nonzero correlation) for each scoring method. A final ranking of the signatures by their correlation with efficacy was obtained by ordering the median rankings across scoring methods.

**References**

1. Estep P, Reid F, Nauman C, Liu Y, Sun T, Sun J, et al. High throughput solution-based measurement of antibody-antigen affinity and epitope binning. *MAbs* (2013) 5(2):270-8. doi:10.4161/mabs.23049

2. Zheng GX, Terry JM, Belgrader P, Ryvkin P, Bent ZW, Wilson R, et al. Massively parallel digital transcriptional profiling of single cells. *Nat Commun* (2017) 814049. doi:10.1038/ncomms14049

3. Li H, van dLAM, Yofe I, Lubling Y, Gelbard-Solodkin D, van AACJ, et al. Dysfunctional CD8 T cells form a proliferative, dynamically regulated compartment within human melanoma. *Cell* (2019) 176(4):775-89 e18. doi:10.1016/j.cell.2018.11.043

4. Hao Y, Hao S, Andersen-Nissen E, Mauck WM, 3rd, Zheng S, Butler A, et al. Integrated analysis of multimodal single-cell data. *Cell* (2021) 184(13):3573-87 e29. doi:10.1016/j.cell.2021.04.048

5. Martin M, Cutadapt removes adapter sequences from high-throughput sequencing reads, EMBnet.journal, 2011.

6. Dobin A, Davis CA, Schlesinger F, Drenkow J, Zaleski C, Jha S, et al. STAR: ultrafast universal RNA-seq aligner. *Bioinformatics* (2013) 29(1):15-21. doi:10.1093/bioinformatics/bts635

7. Li B, Dewey CN. RSEM: accurate transcript quantification from RNA-Seq data with or without a reference genome. *BMC Bioinformatics* (2011) 12323. doi:10.1186/1471-2105-12-323

8. Dennis L, Kaufmann S, Danaher P, Bailey C, Beechem J, Multiplexed cancer immune response analysis, NanoString Technologies®, Inc, 2019.

9. NanoString TI, nCounter® PanCancer IO 360™ Panel, Seattle, WA.

10. Charoentong P, Finotello F, Angelova M, Mayer C, Efremova M, Rieder D, et al. Pan-cancer Immunogenomic Analyses Reveal Genotype-Immunophenotype Relationships and Predictors of Response to Checkpoint Blockade. *Cell Rep* (2017) 18(1):248-62. doi:10.1016/j.celrep.2016.12.019

11. Cristescu R, Mogg R, Ayers M, Albright A, Murphy E, Yearley J, et al. Pan-tumor genomic biomarkers for PD-1 checkpoint blockade-based immunotherapy. *Science* (2018) 362(6411). doi:10.1126/science.aar3593

12. Lee E, Chuang HY, Kim JW, Ideker T, Lee D. Inferring pathway activity toward precise disease classification. *PLoS Comput. Biol* (2008) 4(11):e1000217. doi:10.1371/journal.pcbi.1000217

13. Yamaguchi KD, Ruderman DL, Croze E, Wagner TC, Velichko S, Reder AT, et al. IFN-beta-regulated genes show abnormal expression in therapy-naive relapsing-remitting MS mononuclear cells: gene expression analysis employing all reported protein-protein interactions. *J Neuroimmunol* (2008) 195(1-2):116-20. doi:10.1016/j.jneuroim.2007.12.007

14. Tomfohr J, Lu J, Kepler TB. Pathway level analysis of gene expression using singular value decomposition. *BMC Bioinformatics* (2005) 6225. doi:10.1186/1471-2105-6-225

15. Foroutan M, Bhuva DD, Lyu R, Horan K, Cursons J, Davis MJ. Single sample scoring of molecular phenotypes. *BMC Bioinformatics* (2018) 19(1):404. doi:10.1186/s12859-018-2435-4

16. Barbie DA, Tamayo P, Boehm JS, Kim SY, Moody SE, Dunn IF, et al. Systematic RNA interference reveals that oncogenic KRAS-driven cancers require TBK1. *Nature* (2009) 462(7269):108-12. doi:10.1038/nature08460

17. Hanzelmann S, Castelo R, Guinney J. GSVA: gene set variation analysis for microarray and RNA-seq data. *BMC Bioinformatics* (2013) 147. doi:10.1186/1471-2105-14-7

**Supplemental Figure 1. TIGIT expression on normal peripheral cells versus intra-tumoral lymphocytes.** Dissociated human lung tumors were assessed for expression of TIGIT, both mean fluorescence intensity (MFI) (**A and C**) and percentage of cells positive (**B**), on gated CD8+, CD4+ and Tregs (gated as CD25+FOXP3+). Two donors of normal, healthy PBMCs were included as controls.

**Supplemental Figure 2. Intratumoral changes in CD8, CD4 and Treg populations in response to various anti-TIGIT mAbs. A-C)** CT26 tumors were harvested 1 day post third dose of the indicated anti-TIGIT mAb (1 mg/kg) and analyzed via FACS for infiltrating lymphocytes. % of CD45+ cells that are CD4+ (A), CD8+ (B) and % of CD4+ cells that are Tregs and CD25+CD127- (C) are denoted in the graphs. Each dot represents and individual mouse ± SEM. D-F) MC38 tumors were also harvested 2 days post second dose and analyzed similarly for CD8+ (**D**), CD4+ (**F**) and Tregs (**E**).
