# Supplementary figures and images for: Nonfucosylation of an anti-TIGIT antibody enhances FcγR engagement, driving innate immune activation and antitumor activity
Source: Front Immunol. 2023 Nov 1;14:1280986. doi: 10.3389/fimmu.2023.1280986 (PMC10654636; doi:10.3389/fimmu.2023.1280986)

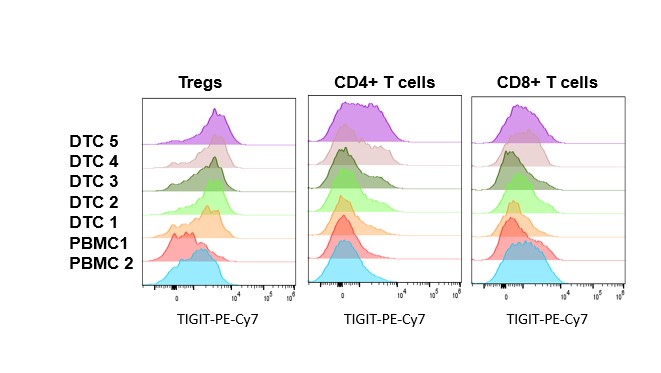

Supplement: Supplementary file 2 [file Image_1.jpeg]

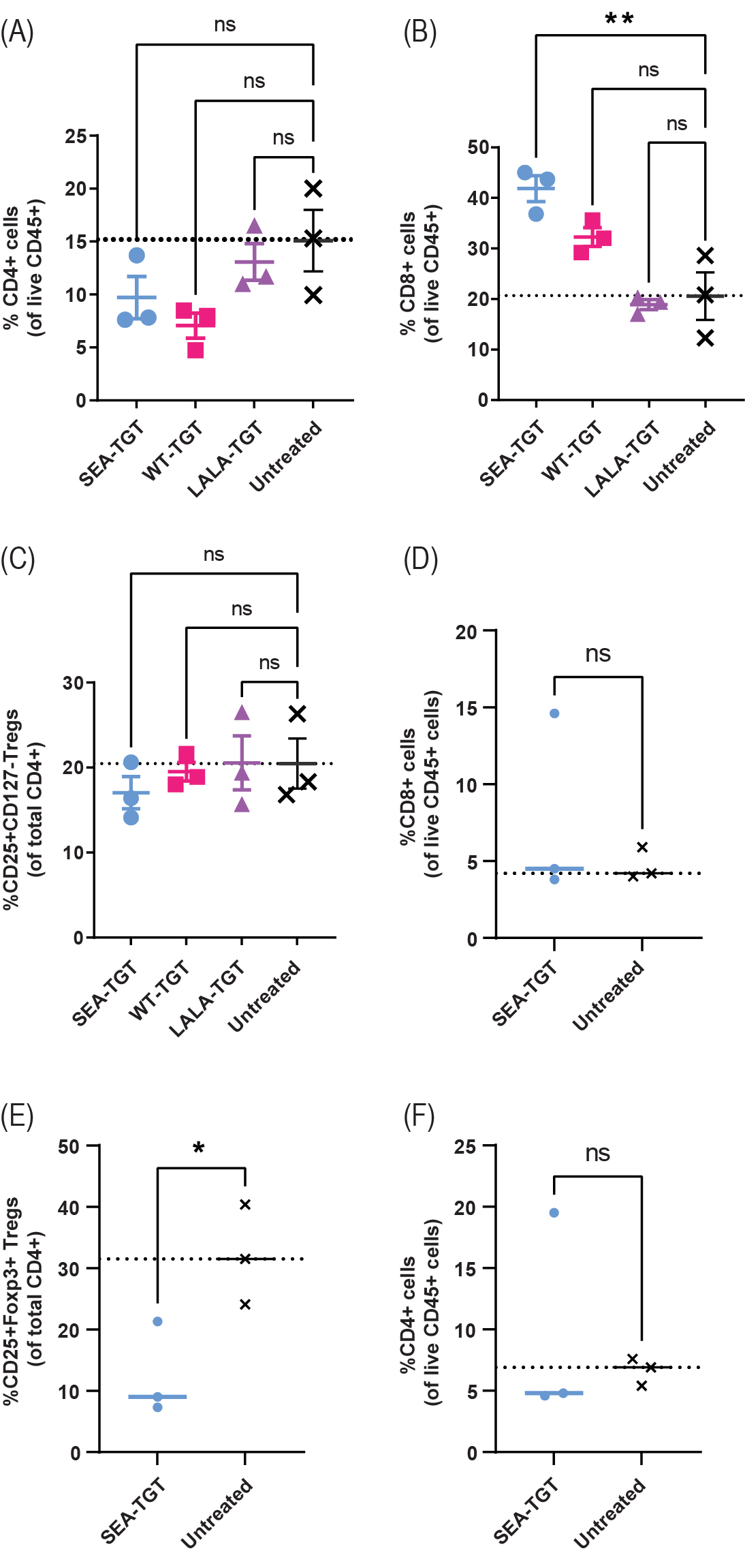

Supplement: Supplementary file 3 [file Image_2.jpeg]
